# Supplementary material for: Cryo-EM structures of the tubulin cofactors reveal the molecular basis of alpha/beta-tubulin biogenesis
Source: Nat Commun. 2025 Dec 29;17:1405. doi: 10.1038/s41467-025-68142-0 (PMC12881605; doi:10.1038/s41467-025-68142-0)
Supplement: Supplementary file 1 — Supplementary Information [file 41467_2025_68142_MOESM1_ESM.pdf]

Cryo-EM structures of the tubulin cofactors reveal the molecular basis of alpha/beta-tubulin biogenesis

Aryan Taheri[1,3], Zhoaqian Wang[1,4], Bharti Singal[1,5], Fei Guo[1], Jawdat Al-Bassam[1]

[1]Molecular Cellular Biology Department, University of California, Davis, CA, USA

Supplementary Figures S1-S22

Supplementary Movies S1-S4

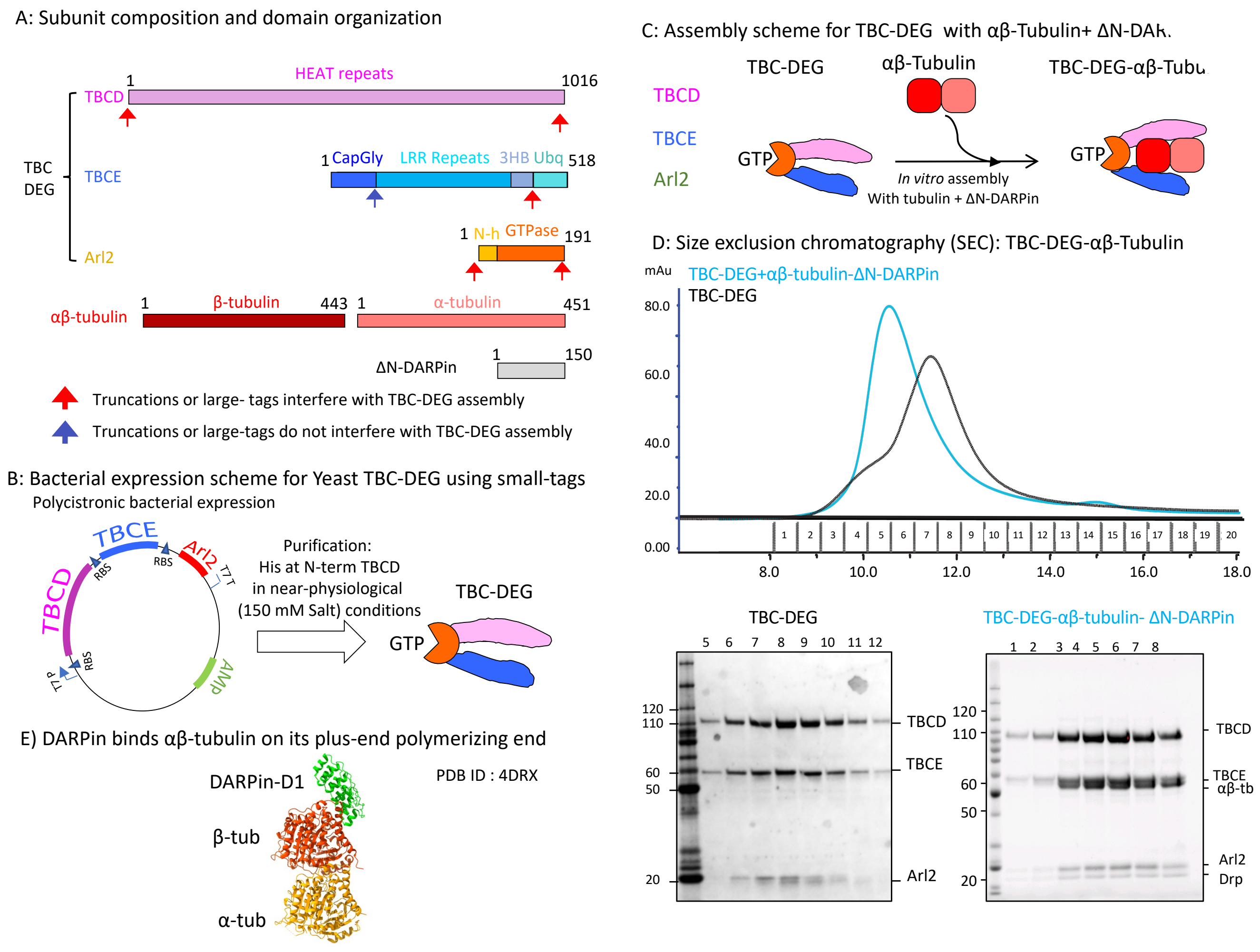

Supplementary Fig 1: **Purification of recombinant TBC-DEG and Reconstitution of the TBC-DEG-αβ-tubulin assemblies for cryo-EM**

A) Subunit and domain organization, and residue length of TBCD, TBCE, Arl2, α-tubulin, β-tubulin, and ΔN-DARPin. Red arrows denote sites where subunit truncations or insertions of large protein tags led to defects in TBC-DEG solubility, blue arrows denote sites where subunit truncations or insertion of large protein tags did not lead to defects in TBC-DEG solubility as described[1].

B) Expression scheme for TBCD, TBCE, and Arl2 purified using a short 6X his tag on the N-terminus of TBCD and purified using *E. coli* bacteria overexpression and purified using physiological ionic conditions.

C) Scheme describing the reconstitution of TBC-DEG with soluble αβ-tubulin to form TBC-DEG-αβ-tubulin assemblies. Note that ΔN-DARPin was utilized to stabilize the αβ-tubulin and prevent TBC-DEG-αβ-tubulin aggregation.

D) Top panel, Size exclusion chromatography (SEC) for TBC-DEG alone (black trace) and TBC-DEG-αβ-tubulin-ΔN-DARPin (blue trace) showing the elution profiles of the assemblies. Bottom panel, left, SDS PAGE showing contents of SEC fractions for TBC-DEG alone including TBCD, TBCE, and Arl2 subunits. Right, SDS PAGE showing fractions of TBC-DEG-αβ-tubulin for SEC fraction of TBC-DEG/αβ-tubulin showing the contents of TBCD, TBCE, Arl2, α-tubulin, β-tubulin, and ΔN-DARPin.

E) The binding interface for ΔN-DARPin to the plus-end polymerizing interface of β-tubulin as observed in previous structures.

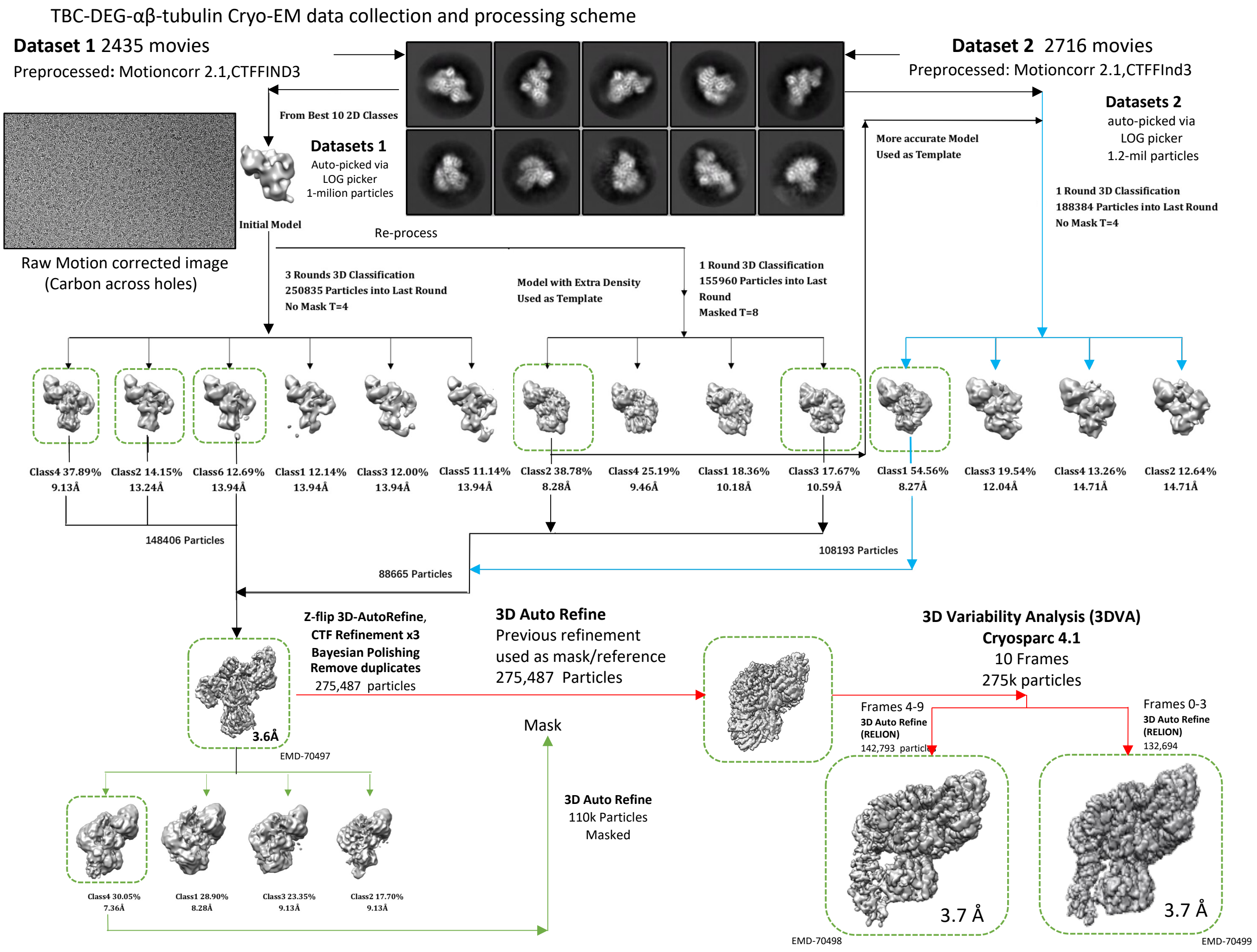

Supplementary Fig 2: Cryo-EM image data collection and single particle image process for TBC-DEG-αβ-tubulin complexes leading to structures of two unique states (class 1 and class 2).

Top to bottom, two TBC-DEG-αβ-tubulin datasets (top left and top right; example image shown in the middle) were collected and raw images were pre-processed using motioncorr2, CTFFind3 then used to identify coordinates for particle images, which were processed using a combination of RELION 3-4.0 and cryosparc 3.0-4.1. Multiple cycles of 3D classification and 3D refinement led to 3.6-Å TBC-DEG-αβ-tubulin core particle with low resolution for a mobile arm-like extension (described in more detail in Supplementary Fig 3). The conformation of the arm-like extension was resolved using 3DVA analysis using a mask around the arm region leading two unique classes (State 1 and State 2) in which TBCE conformation was unique. Final processing statistics are described in Table I.

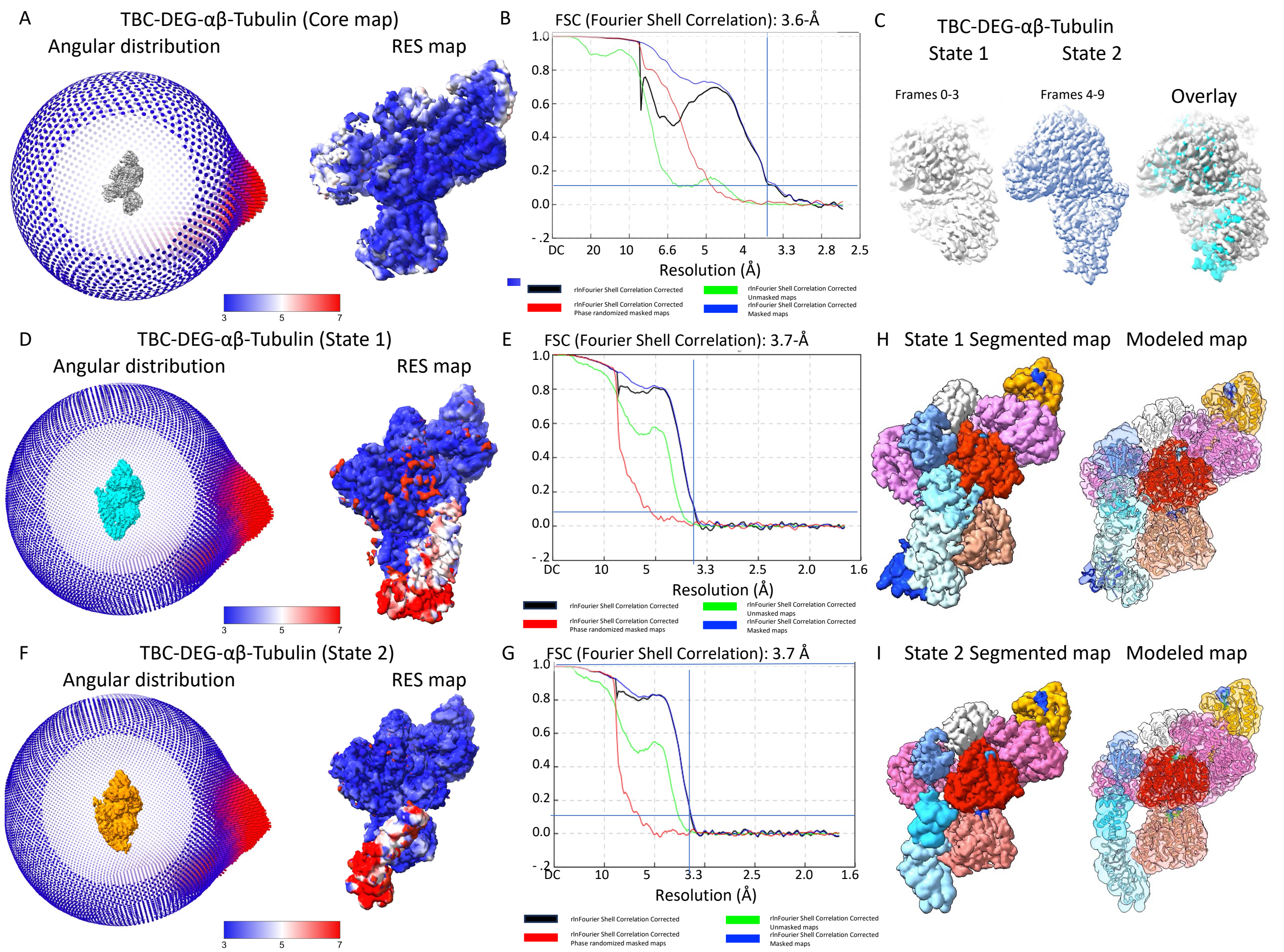

Supplementary Fig 3: **Details of the 3D variability (3DVA) analysis of TBC-DEG-αβ-tubulin cryo-EM maps leading to the two TBCE conformations bound to α-tubulin.**

A) Left, Angular distribution for the TBC-DEG-αβ-tubulin Core region particles in the final refined map. Middle panel, Resmap for TBC-DEG-αβ-tubulin showing the resolution distribution color range onto the structure.
B) Gold standard Fourier Shell correlation (FSC) for TBC-DEG-αβ-tubulin core map.
C) Comparison of TBC-DEG-αβ-tubulin State 1 and State 2 refined cryo-EM maps. Left panel, refined state 1 TBC-DEG-αβ-tubulin cryo-EM map (frames 0-3). Middle panel, refined state 2 TBC_DEG-αβ-tubulin cryo-EM map (frames 7-10). Right panel, overlay of both maps showing the conformation change in the Arm-like extension representing TBCE.
D) Left, Angular distribution for TBC-DEG-αβ-tubulin State 1 particles in the final refined map. Middle panel, Resmap for TBC-DEG-αβ-tubulin in State 1 showing the resolution distribution color range onto the structure.
E) Left, Angular distribution for TBC-DEG-αβ-tubulin State 2 particles in the final refined map. Middle panel, Resmap for TBC-DEG-αβ-tubulin in State 2 showing the resolution distribution color range onto the structure.
F) Gold standard Fourier Shell correlation (FSC) for TBC-DEG-αβ-tubulin state 1.
G) Gold standard Fourier Shell correlation (FSC) for TBC-DEG-αβ-tubulin state 2.
H) Left, Model-based segmented TBC-DEG-αβ-tubulin-ΔN-DARPin state 1 map (segmented map), right, atomic models for subunits placed in segments of these subunits of TBC-DEG-αβ-tubulin-ΔN-DARPin.
I) Left, Model-based segmented TBC-DEG-αβ-tubulin-ΔN-DARPin state 2 map (segmented map), right, atomic models for subunits placed in segments of these subunits of TBC-DEG-αβ-tubulin-ΔN-DARPin (modeled map).

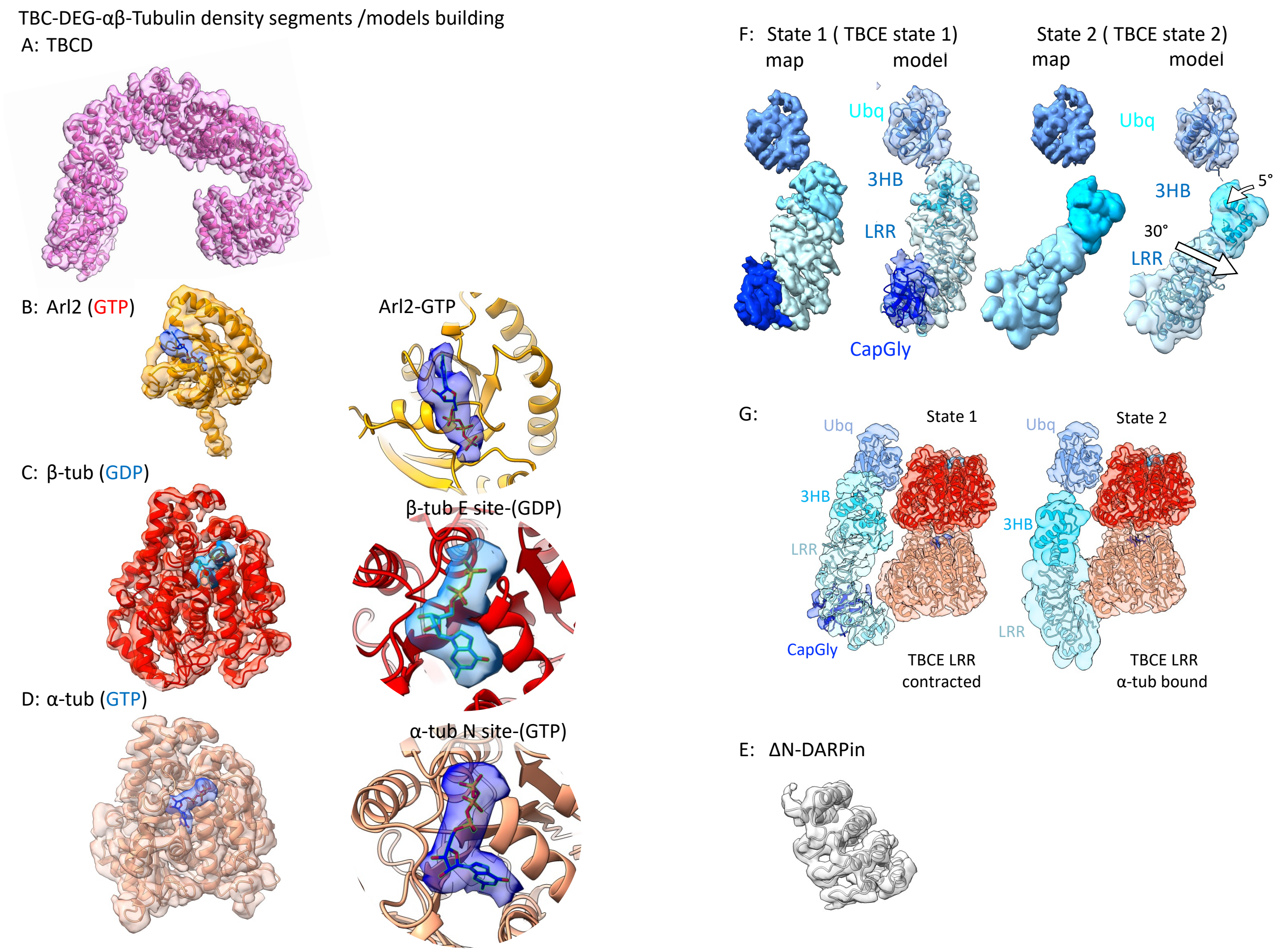

Supplementary Fig 4: **Cryo-EM map segments and atomic models built of each of the TBC-DEG-αβ-tubulin subunits including two unique TBCE models and details of the unique TBCE-αβ-tubulin interfaces in the two states.**

A) Map segment (surface) and model built for TBCD (ribbon).

B) Left panel, map segment (surface) and model built for Arl2 (ribbon) with the GTP nucleotide density and model GTP shown in blue. Right panel close-up view for GTP in Arl2 with electron density shown in blue.

C) Left panel, map segment (surface), and model built for β-tubulin (ribbon). Right panel close-up view for E-site GDP in β-tubulin with electron density shown in cyan.

D) Left panel, map segment, and model built for α-tubulin. Right panel close-up view for N-site GTP in α-tubulin with electron density shown in blue.

E) Left panel, map segment, and model built for ΔN-DARPin. Right panel, example density for an atomic model built into the ΔN-DARPin electron density.

F) Left panels, State 1 TBCE map segments showing density map (left), model fitted in subregions of the segmented map (right). Right panels, State 2 TBCE map segments showing segmented density map (left), model fitted into subregion segmented map (right).

G) Left panels, State 1 vs State 2 map comparison showing a slice view of TBCE-αβ-tubulin interface showing the State 1 TBCE retracted state (State 1) versus the TBCE-α-tubulin bound state (state 2). Right panels, State 1 (left) vs State 2 (right) density map viewed with the αβ-tubulin interaction interfaces marked by the color of α-tubulin (light red) and β-tubulin (dark red) showing changes in the TBCE LRR in interfacing with α-tubulin in State 2 compared to State 1.

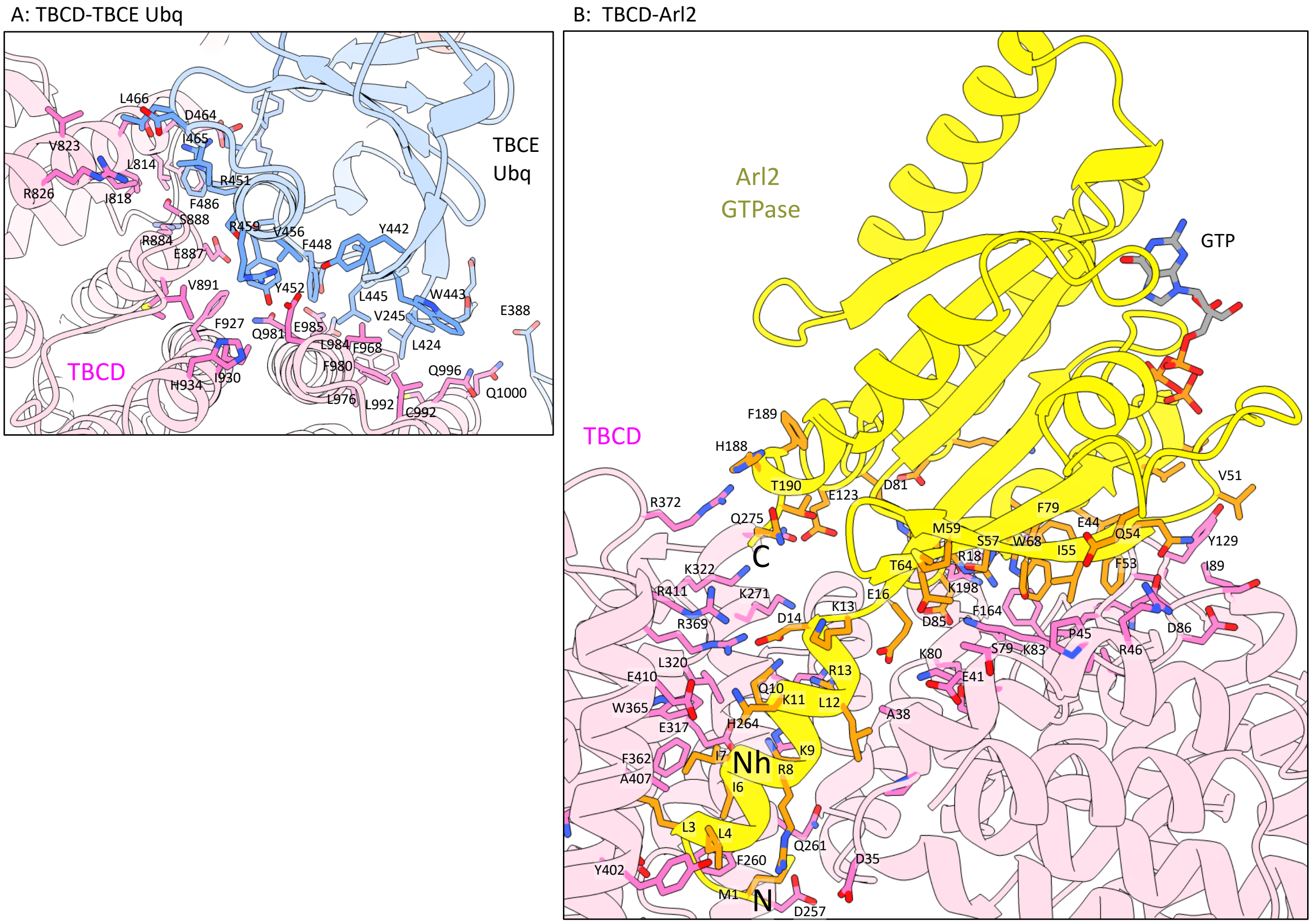

Supplementary Fig 5: Atomic interaction views of Arl2, TBCE binding to TBCD to form the TBC-DEG assembly as viewed in PDB-ID 9DER:

A) Top view of the TBCE Ubq (cyan) -TBCD spiral (pink light) interface, with residues shown in darker colors. Note the mixture of the ionic and aliphatic residue interactions in holding the TBCE interface.

B) Side View of Arl2 TBCD interface showing the Arl2 Nh (light orange) embedded in TBCD turret (light pink) and the Arl2 GTPase (light orange)-TBCD turret (light pink) interface, with interacting residues shown in heteroatom and darker subunit colors. Note the TBCD turret binds Arl2 NH using charged ionic (Q, N, R and K residues binding Q, N, D and E) networks while binds the GTPase domain using Aliphatic residues (Y, W and F residues). Sequence conservations, presented in Supplementary Fig 17-18, and their plots on the structures, in Supplementary Fig 20, provide additional information.

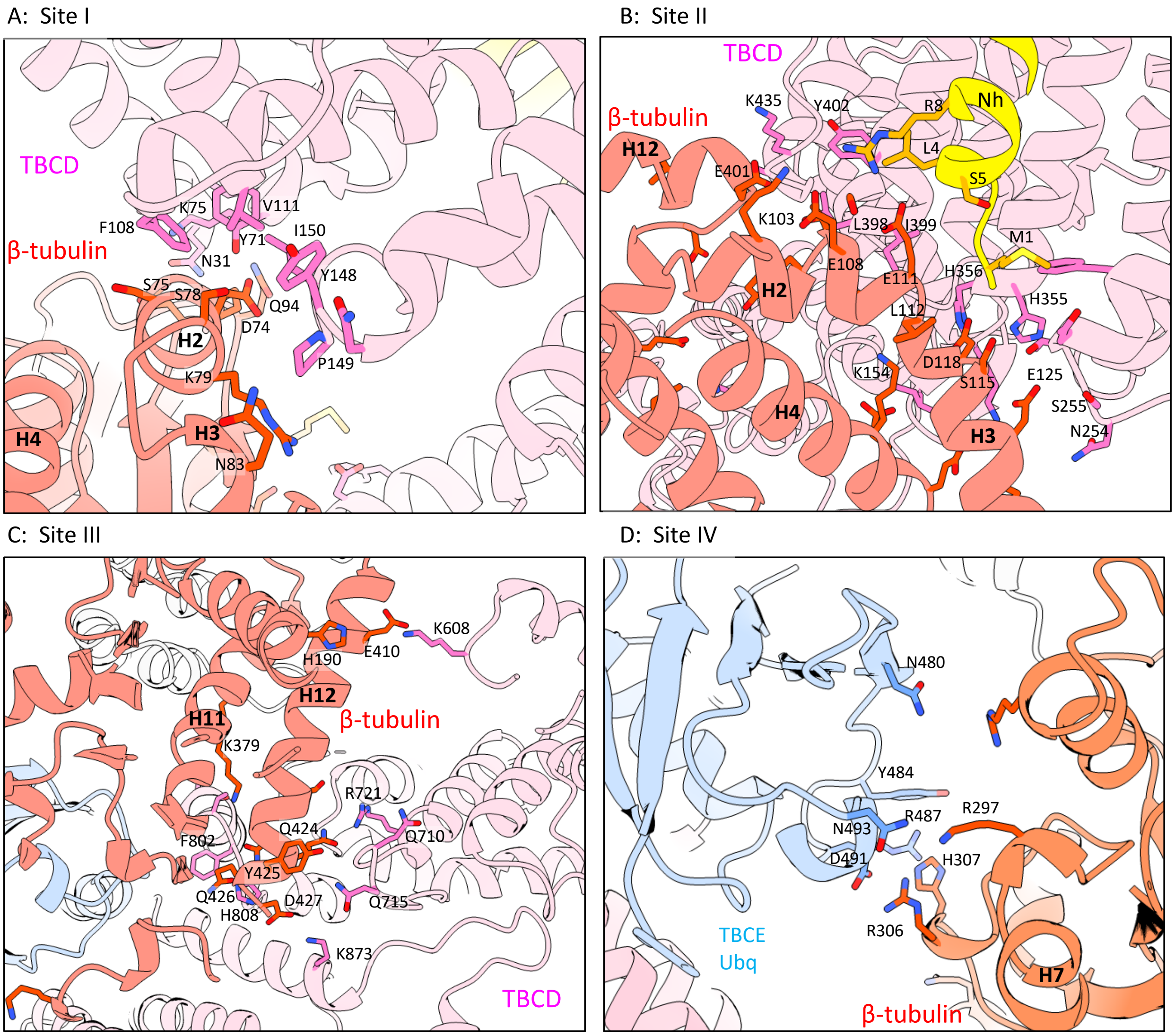

Supplementary Fig 6: atomic view of the TBC-DEG -β-tubulin interaction interfaces.

The interactions are supported by AlphaFold3 interfaces in Supplementary Fig 7. Sequence conservations, presented in Supplementary Fig 17-18, and their plots on the structures, in Supplementary Fig 20, provide additional information.

A) TBC-DEG interface I on β-tubulin. TBCD turret (light pink) interacts with β-tubulin (light red). This interface involves TBCD HEAT 1-3 helices binding β-tubulin H2 and H3.

B) TBC-DEG interface 2 on β-tubulin. TBCD turret (light pink) HEAT 8-10 and Nh of Arl2 (orange) interaction with β-tubulin (light red) H2 and H3 is shown, with residues shown in darker colors.

C) TBC-DEG interface 3 on β-tubulin. TBCD (pink) HEAT 18-20 interacting with β-tubulin (red) H11, H12, and C-terminal tail is shown, with residues shown in darker colors.

D) TBC-DEG interface 4 on β-tubulin. TBCE Ubq (cyan) bound on the TBCD spiral (pink) interacting with β-tubulin (red) H6 and H7 is shown, with residues shown in darker colors.

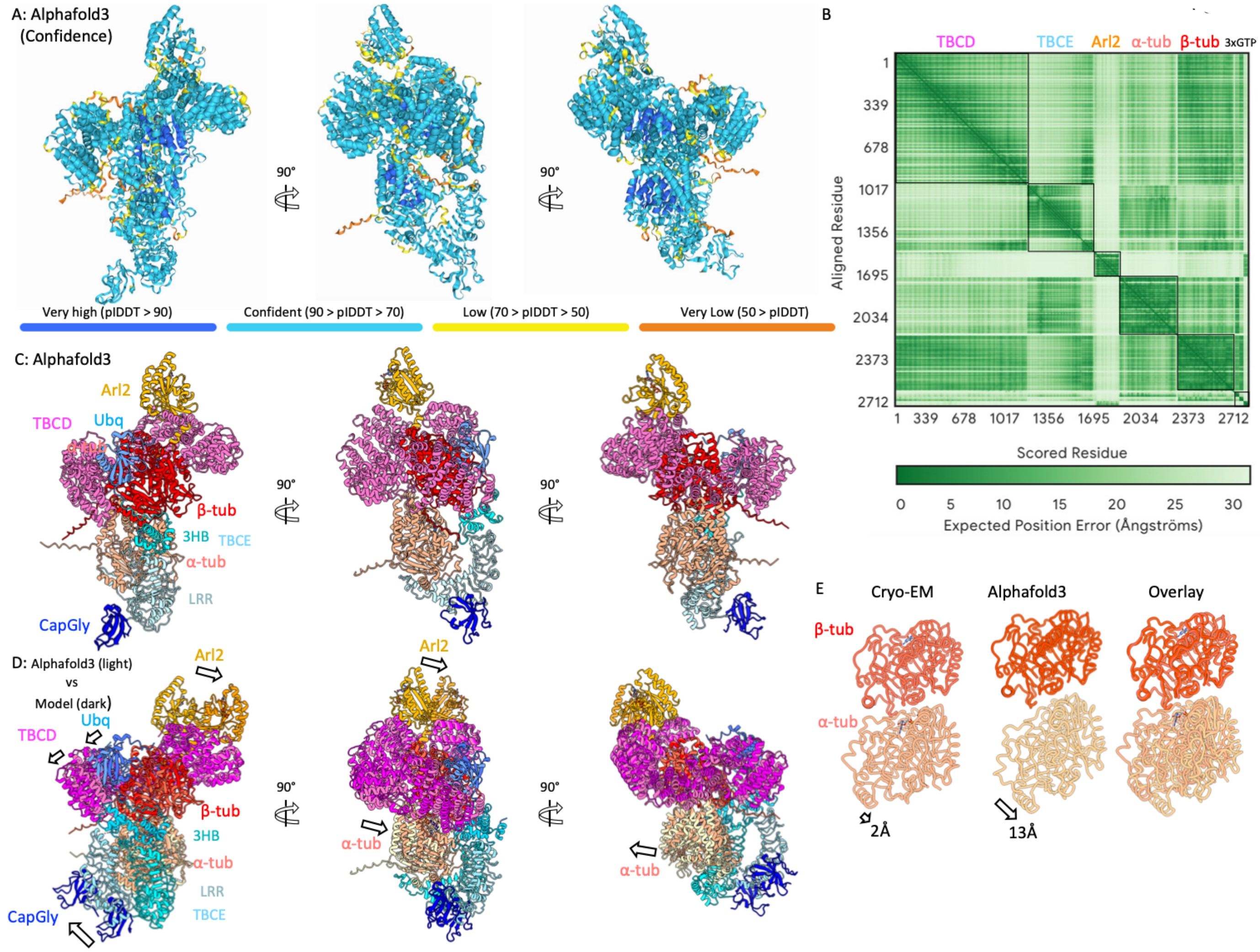

Supplementary Fig 7: AlphaFold3 model for TBC-DEG:αβ-tubulin matches the TBC-DEG:αβ-tubulin cryo-EM model

A) Three rotated views of the TBC-DEG-αβ-tubulin AlphaFold3 model in ribbon format colored based on the IDTT confidence scores following the scale in the lower panel

B) Predicted aligned Error (PAE) matrix plot for TBC-DEG:αβ-tubulin AlphaFold3 prediction including TBCD, TBCE, Arl2, α- and β-tubulin and GTP nucleotides.

C) Three rotated views of the TBC-DEG-αβ-tubulin with the subunits and domains colored as shown in Fig. 1.

D) Three rotated views of the superimposed TBC-DEG-αβ-tubulin cryo-EM model (dark colors) and AlpahFold3 model (light colors). With regions of difference marked with arrows. Note the rotation in Arl2 GTPase and difference in the TBCE arm binding to α-tubulin.

E) Comparison of the αβ-tubulin conformations; cryo-EM model (left), AlphaFold3 (middle) and overlay of both (right). Note the increase in twist in α-tubulin observed in the AlphaFold3 model compared to the cryo-EM model.

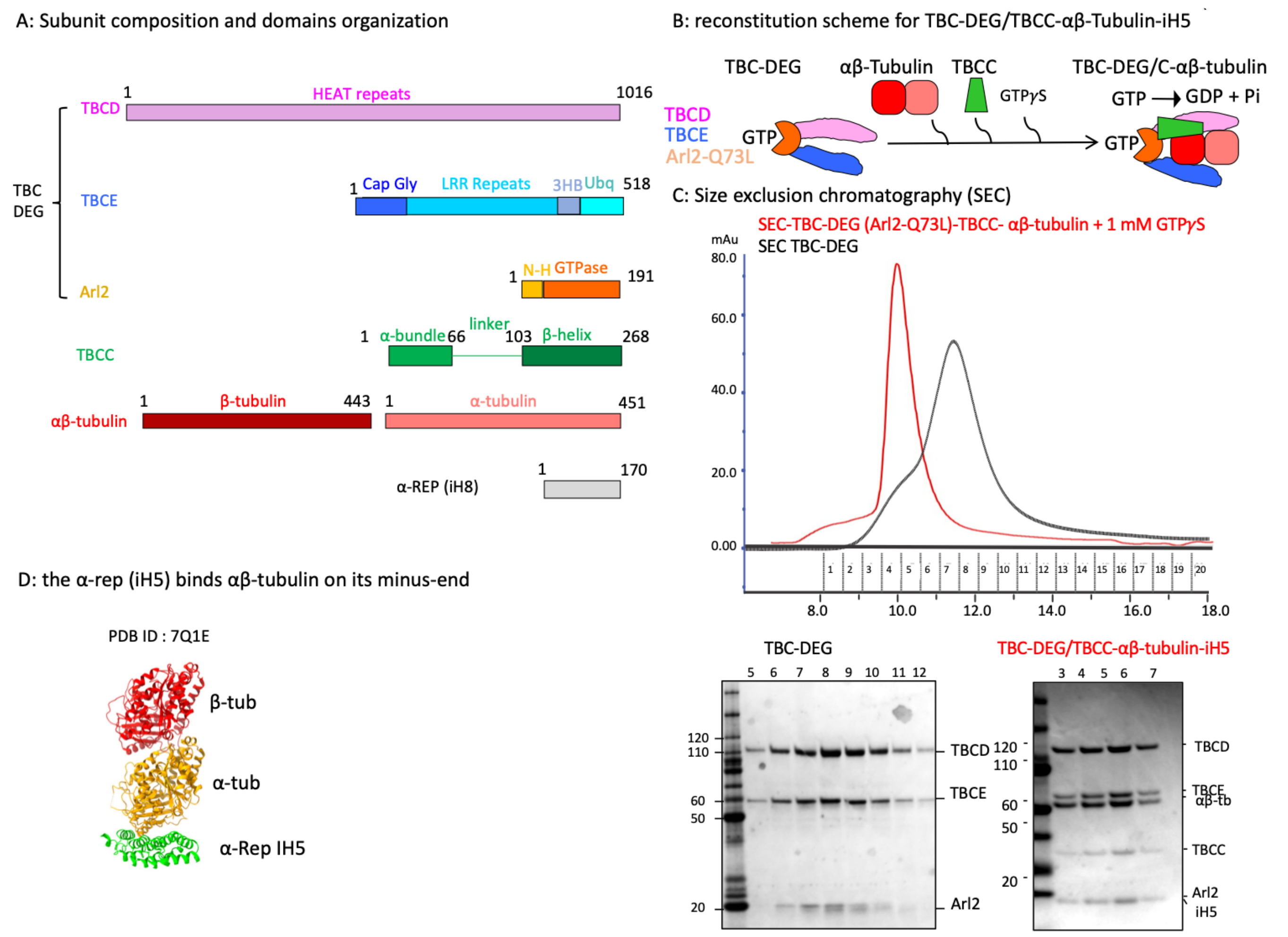

Supplementary Fig 8: **Biochemical reconstitution of TBC-DEG/TBCC-αβ-tubulin assemblies using Arl2 GTP-locked mutant and GTPγS**

A) Subunit and domain organization, and residue length of TBCD, TBCE, Arl2, TBCC, α-tubulin, β-tubulin, and α-rep (iH5).

B) Scheme describing the reconstitution of TBC-DEG-Arl2 Q73L (GTP-locked mutant) with soluble αβ-tubulin and TBCC in the presence of GTPγS to form TBC-DEG/TBCC-αβ-tubulin assemblies. Note that α-rep iiH5 -DARPin was utilized to stabilize the αβ-tubulin in the assembly and prevent aggregation.

C) Top panel, Size exclusion chromatography (SEC) for TBC-DEG alone (black trace) and TBC-DEG-Arl2 Q73L-TBCC-αβ-tubulin-GTPγS-iiH5 (red trace) showing the elution profiles of the assemblies. Bottom panel: left, SDS-PAGE showing SEC fraction contents for TBC-DEG alone including TBCD, TBCE, and Arl2 subunits. Right, SDS-PAGE showing fractions of TBC-DEG-αβ-tubulin for SEC fraction of TBC-DEG/αβ-tubulin showing the contents of TBCD, TBCE, Arl2, TBCC, α-tubulin, β-tubulin and iH5 subunits.

D) The binding interface for iH5 to the minus-end polymerizing interface of α-tubulin as observed in previously determined structures. Note this is the opposite longitudinal surface of αβ-tubulin from ΔN-DARPin which did not bind the TBC-DEG/TBCC-αβ-tubulin assembly.

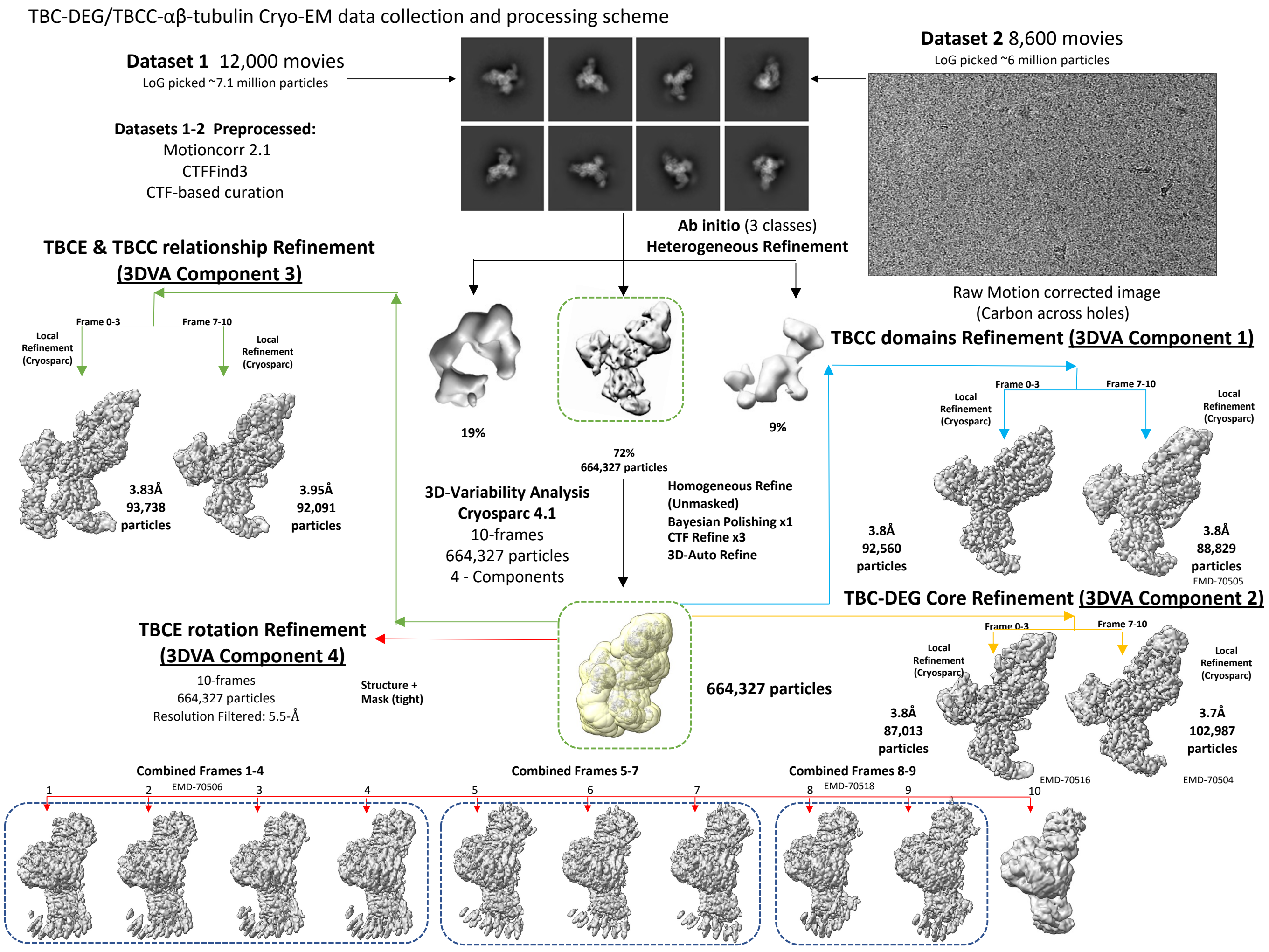

Supplementary Fig 9: **Cryo-EM image data collection and image process for TBC-DEG-αβ-tubulin complexes leading to structures leading to multiple structures of TBC-DEG/TBCC-αβ-tubulin**

Top to bottom, two TBC-DEG/TBCC-αβ-tubulin-iiH5 datasets (top left, top right); example image shown in the top right) were collected and raw images were pre-processed using motioncorr2, CTFfind3 then used identify coordinates for particle images, which were processed using a combination of RELION 3-4 and cryosparc 3-4.1 leading to 2D-class averages which are distinct from TBC-DEG-αβ-tubulin shown in Supplementary Fig 2. A cycle of *ab initio* model generation followed by heterogeneous refinement was carried out using a TBC-DEG-αβ-tubulin core map, leading to 72% of particles remaining in a single moderate resolution class. 3D-refinement led to moderate resolution TBC-DEG/TBCC-αβ-tubulin with multiple blurred low-resolution regions. Four 3DVA components, using cryosparc 4.1, were carried out on the consensus refined TBC-DEG/TBCC-αβ-tubulin particles. TBCC domains were resolved using 3DVA Component 1 (middle right) leading to two classes in which the three TBCC domains are present or absent. The conformational changes in the TBC-DEG/TBCC-αβ-tubulin core assembly were resolved using 3DVA Component 2 (the lower right) leading two classes in which TBCC-induced conformational changes are present or absent in the TBCD and TBCE Ubq domain. The relationship between TBCE and TBCC-N density binding to the TBC-DEG/TBCC-αβ-tubulin core was resolved using 3DVA Component 3 (middle left). The rotational transition of TBCE arm-like extension was resolved using 3DVA component 4 leading to 10 frames with three distinct states for the TBCE arm.

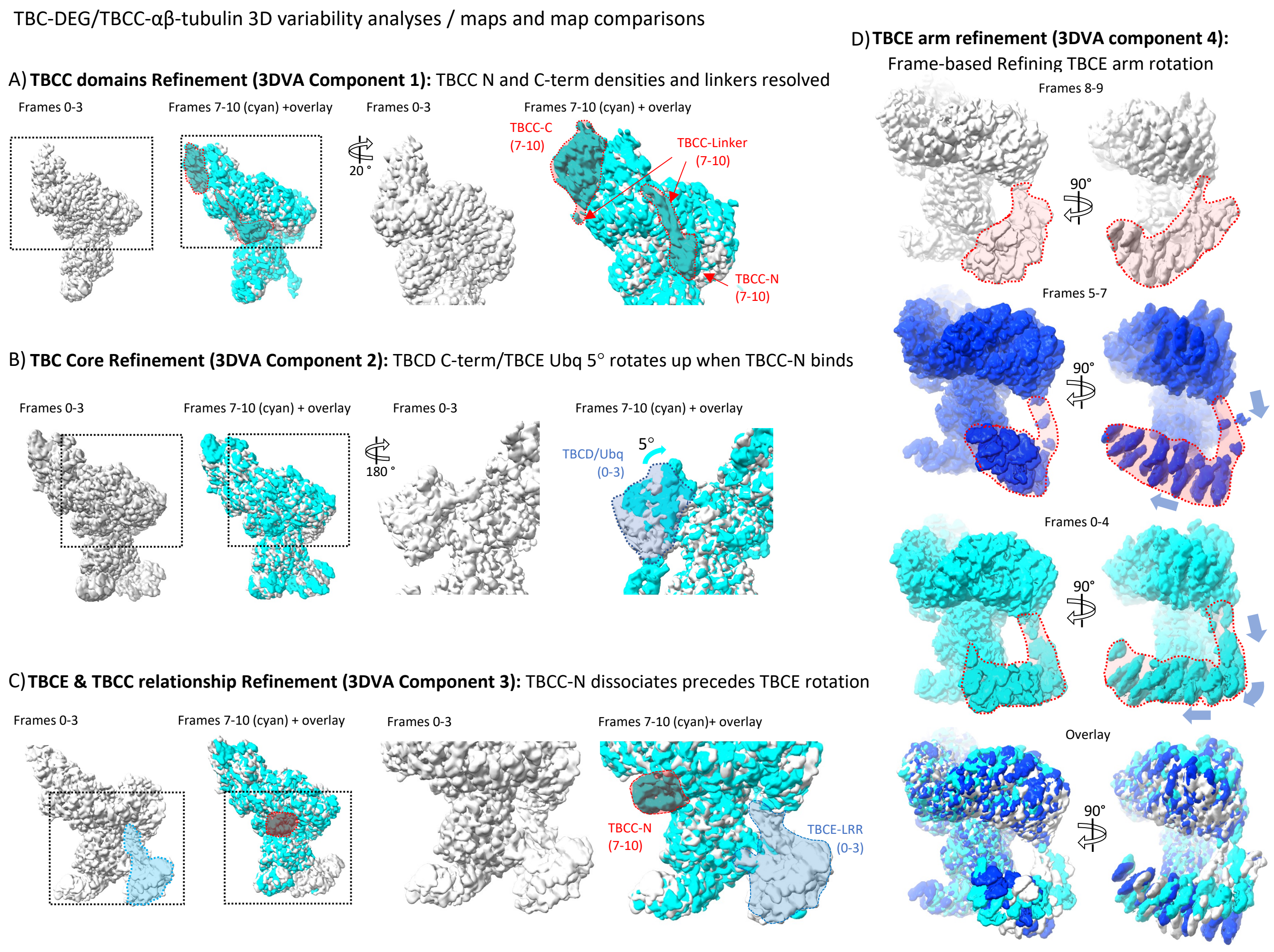

Supplementary Fig 10: **Close-up Comparison of multiple states resolved using four component 3DVA**.

Each 3DVA component led to two distinct states of TBC-DEG/TBCC-αβ-tubulin assemblies and allowed us to generate states with refined maps for different TBC-DEG/TBCC-αβ-tubulin subregions.

A) Comparison of two states resolved in 3DVA component 1: Left Panels: frames 0-3 without TBCC domain (light gray, left), and overlay of frames 7-10 (cyan)+ frames 0-3 (gray) with TBCC domains (highlighted by dotted lines). Right panels, close-up 20° rotated views of right panels: frames 0-3 (light gray), and overlay of frames 7-10 (cyan) and frames 0-3 (light gray) with TBCC-C, TBCC-L, and TBCC-N densities in frames 7-10 (dotted red lines)

B) Comparison of two states resolved in 3DVA component 2 leading to a 5° rotation in the TBCD spiral/TBCE Ubq. Left panels: frames 0-3 with TBCD Spiral /TBCE Ubq in downward rotation (gray) and overlay of frames 7-10 (cyan) with frame 0-3 (gray) showing wTBCD Spiral/TBCE Ubq in the upward a 5° rotation. Right panels, close-up view of a 180°-rotated view of frames 0-3 (gray), overlay of frames 7-10 (cyan) with frames 0-3 (gray) with region rotating marked (black dotted line).

C) Comparison of the two states revolved in 3DVA component 3 leading to a relationship between TBCC-N binding and TBCE arm rotation conformation. Left panels, frames 0-3 (gray) compared to an overlay of frames 7-10 (cyan) and frame 0-3 (gray) showing TBCE being ordered (highlighted in blue) if TBCC-N density is missing (marked red). Right panels, view showing frames 0-3 (gray) compared to overlay of frames 7-10 (cyan) with frames 0-3 (light gray). These maps describe the relationship of the TBCC-N binding to the TBCE arm-like extension.

D) Close-up comparison of three states resolved in 3DVA component 4 leading two extreme states of TBCE LRR-CapGly arm. Top panel, two views of frames 8-9 (light gray) showing the two views of the TBCE arm. Second panels, identical views of frames 5-7 (dark blue). Third panels. Identical views of frames 0-4 (cyan) of TBCE LRR-CapGly Arm-like extension showing its conformational transition and rotation. The bottom panels, two views of all three states shown in panels above demonstrating the tilt, and rotation of the TBCE LRR-CapGly arm-like extension compared to TBC-DEG core.

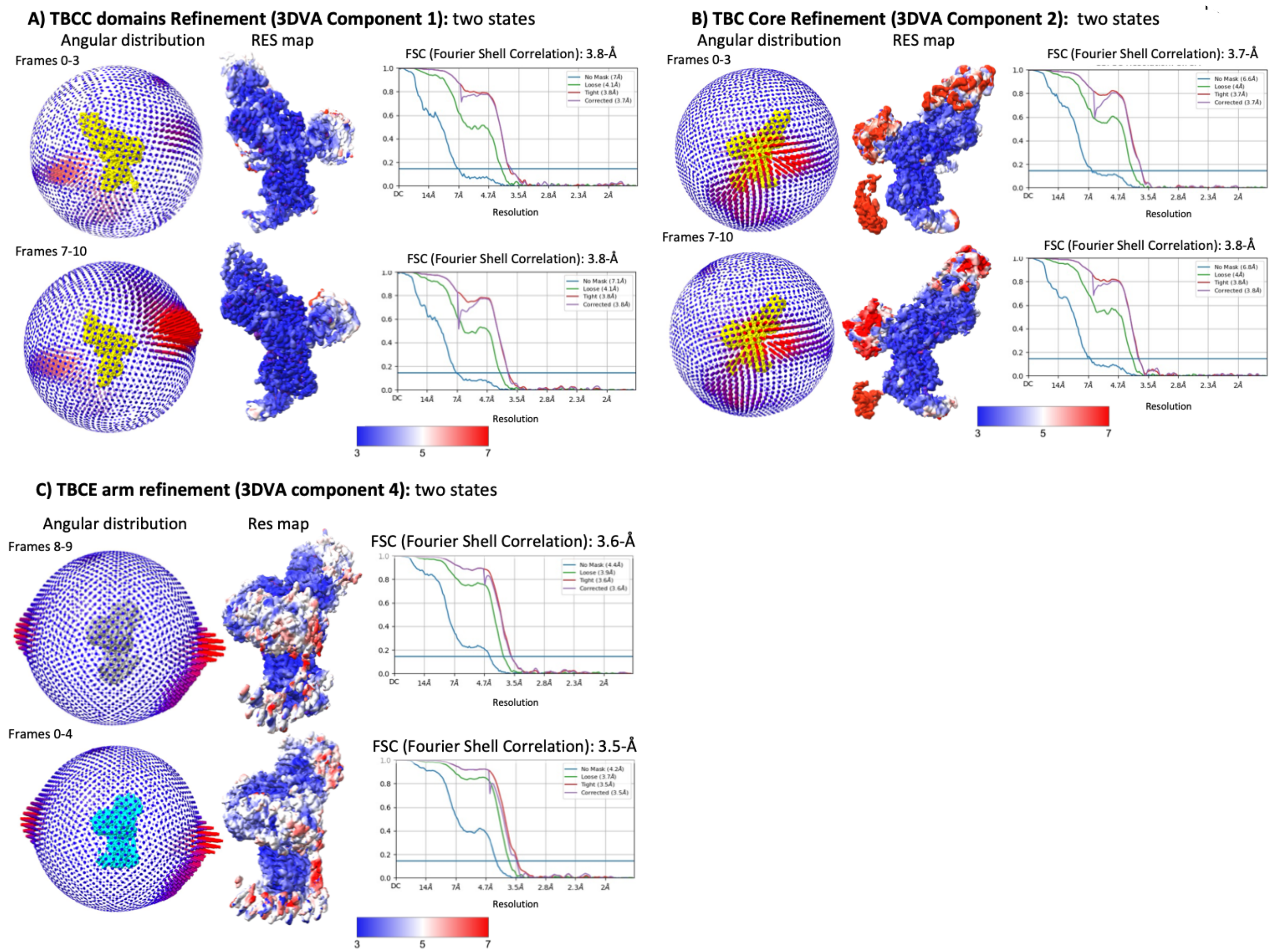

Supplementary Fig 11: **Details of the 3DVA maps used to generate two unique composite states of TBC-DEG/TBCC-αβ-tubulin states.**

A) Two maps from 3DVA component 1 refining TBCC-Domains: Top panels, Frames 0-3 and Bottom panels, Frames 7-10; Left panels, Angular distribution for particles in the final refined map. Middle panels, Res-map showing the resolution distribution color range onto the structure. Right panels, Fourier Shell Correlation (FSC) for each map.

B) Two maps from 3DVA component 2 refining the TBC-DEG core states: Top panels, Frames 0-3 and Bottom panels, Frames 7-10; Left panels, Angular distribution for particles in the final refined map. Middle panels, Res-map showing the resolution distribution color range onto the structure. Right panels, Fourier Shell Correlation (FSC) for each map.

C) Two maps from 3DVA component 4 refining the TBCE arm conformation. Top panels, Frames 8-9 and Bottom panels, Frames 0-4; Left panels, Angular distribution for particles in the final refined map. Middle panels, Res-map showing the resolution distribution color range onto the structure: right panels, Fourier Shell Correlation (FSC) for each map.

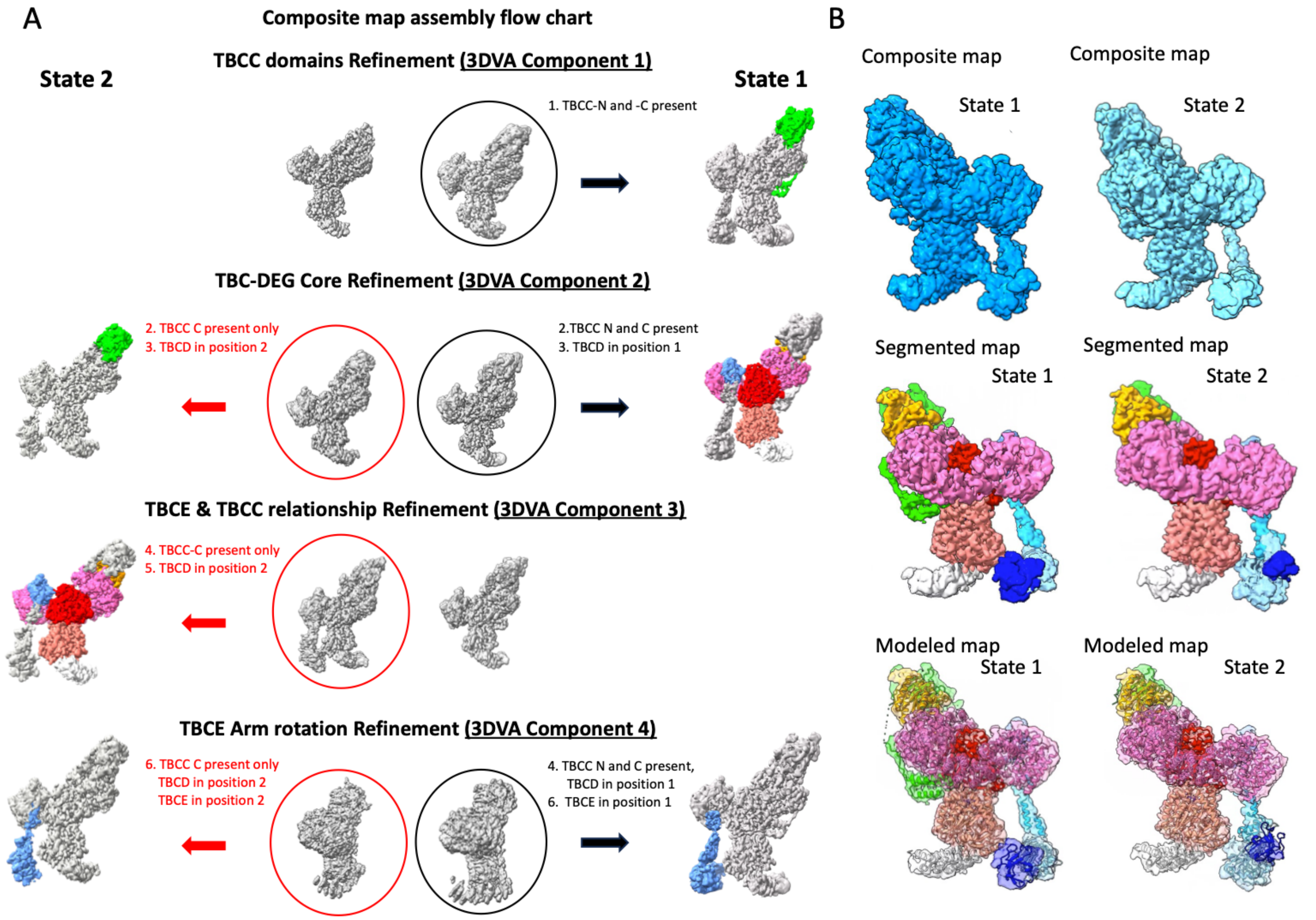

Supplementary Fig 12: Scheme for assembly of the TBC-DEG/TBCC-αβ-tubulin composite maps for state 1 and State 2 using observations in 3DVA components 1-4 maps.

A) Flow chart for the assembly of state 1 and state 2 composite maps from the four component 3DVA analysis maps. The components highlighted in color are utilized from maps for each 3DVA components. Top panel, 3DVA component 1 resolved the full density for TBCC-N and TBCC-C domains in state 1. Second panel, 3DVA component 2 resolved the TBCC-C state 2 (right) and the TBCD, TBCE-Ubq αβ-tubulin for state 1 (left). Third panel,3DVA component 3 resolves the TBCD, TBCE-Ubq αβ-tubulin for state 2 (right). Fourth panel, 3DVA component 4 resolves the two TBCE arm maps for state 1 (left) and state 2 (right).

B) Composite maps for TBC-DEG/TBCC-αβ-tubulin state 1 (state 1) are shown top left, and TBC-DEG/TBCC-αβ-tubulin state 2 (state 2) on top right. Second panels show the Segmented maps for state 1 and state 2 left and right, respectively. Third panel, modeled maps for in segmented maps for state 1 and state 2 are shown on the left and right, respectively.

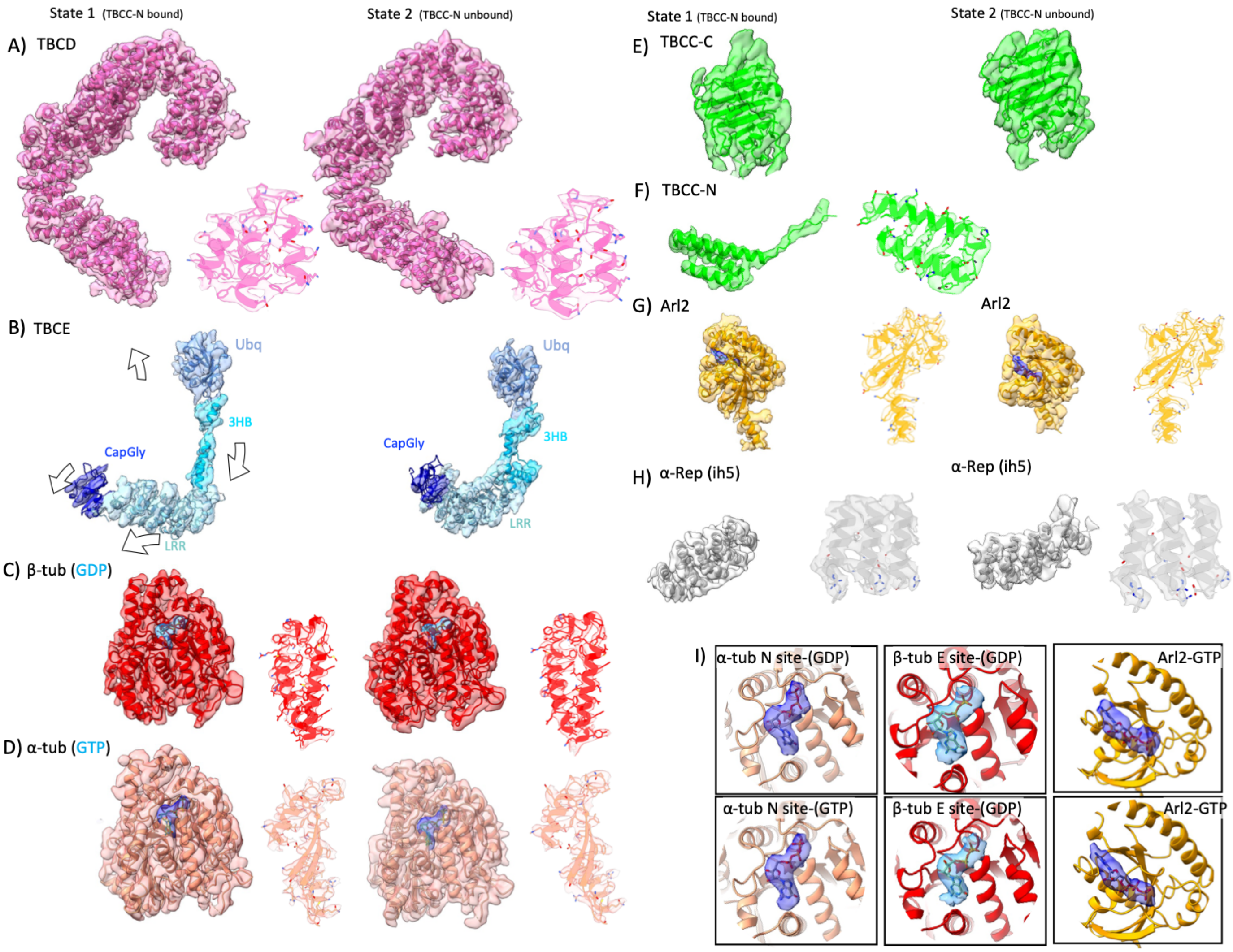

Supplementary Fig 13: **Cryo-EM map segments and atomic models built of each of the TBC-DEG/TBCC-αβ-tubulin subunits.** The segments with built models of the state 1 (TBCC-N bound) (left, panel) and state 2 (TBCC-N unbound) (middle panel) and overlay of the two-state models (right panel) showing the conformational changes in various subunits

A) Map segment and model for TBCD State 1 (pink, left panels), TBCD State 2 (pink, right panel). Close-up electron density panels show example next to full segment panel.

B) Map segment and model for TBCE State 1 (colored according to subdomains, left panel), TBCE State 2 (colored according to subdomains middle panel). Conformational changes are marked on various domains. These regions are of low resolution (6-8Å)

C) Map segment and model for β-tubulin State 1 (red, left panel), β-tubulin State 2 (red, middle panel), Close-up electron density panels show example next to full segment panel.

D) Map segment and model for α-tubulin State 1 (left panel), α-tubulin State 2 (right panel). Close-up electron density panels show example next to full segment panel.

E) Map segment and model for TBCC-C state 1 (lime green, left panel), β-tubulin state 2 (lime green, middle panel). These regions are lower resolution (5Å).

F) Map segment and model for TBCC-N (lime green). Close-up electron density panels show example next to full segment.

G) Map segment and model for Arl2 state 1 (orange, left panel), β-tubulin state 2 (orange, right panel). Close-up electron density panels show example next to full segment panel.

H) Map segment and model for α-Rep iiH5 in state1 (Grey, left panel), α-Rep iH5 in state2 (light gray middle panel), and overlay of the two models (right panel).

I) Close-up views of β-tubulin E-site (GTP), α-tubulin N-site (GDP), and Arl2 (GTP) nucleotide densities in State 1 (top panels) and State 2 (bottom panels) comparable to those displayed for the TBC-DEG-αβ-tubulin map shown in Supplementary Fig 4.

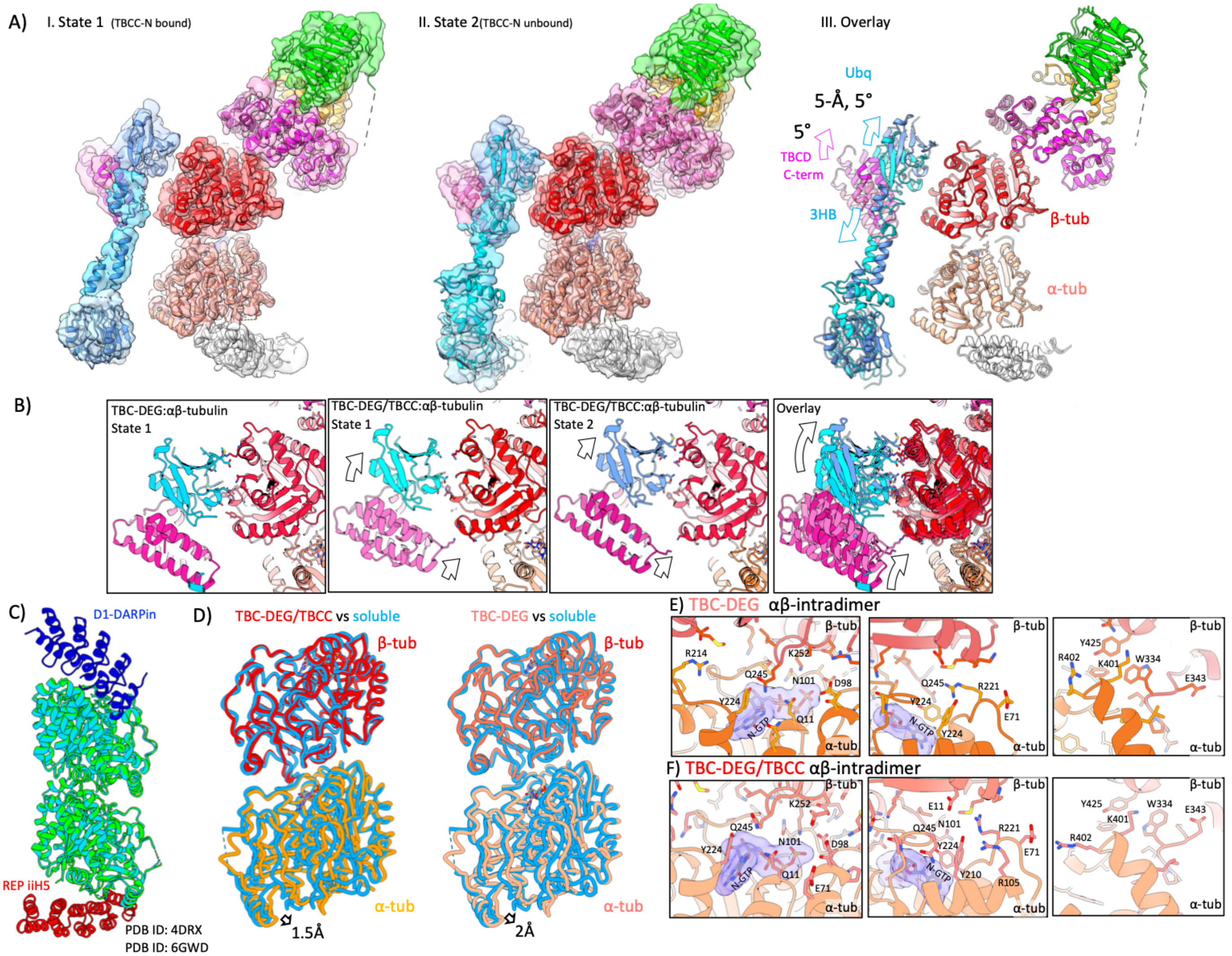

Supplementary Fig 14: **Additional views of TBC-DEG/TBCC-αβ-tubulin conformational changes in TBCD, TBCE, and their interactions with β-tubulin.**

A) Side slice view of TBC-DEG/TBCC-αβ-tubulin focusing on TBCE Ubq, 3HB and TBCD C-terminal domain interacting with β-tubulin; Left panel (I.) state 1 (TBCC-N bound); middle panel (II.), state 2 (TBCC-N unbound). Right panel (III.), overlay of model for state 1 and state 2, marking conformational changes in TBCD and TBCE.

B) Close up view of the TBCD TBCE Ubq β-tubulin interface in three TBC-DEG cryo-EM models. Left panel (I.) TBC-DEG-αβ-tubulin state 1, second from left (II.) TBC-DEG-αβ-tubulin state 1, third from left (III.) TBC-DEG-αβ-tubulin state 2. Right panel (IV). Overlay of all four states showing the gradual transition of TBCE Ubq and TBCD C-terminal domains with respect to β-tubulin.

C) Comparison of TBC-DEG bound αβ-tubulin conformation to soluble αβ-tubulin structures. Left panel, DARPin (blue) and iiH5 (green) bound αβ-tubulin crystal structures overlaid; middle panel, overlay of DARPin bound soluble αβ-tubulin (blue) and TBC-DEG/TBCC bound αβ-tubulin (brown). Right panel, overlay of DARPin bound soluble αβ-tubulin (blue) and TBC-DEG bound αβ-tubulin (teal). Note the α-tubulin rotation in right panel. Comparisons are mode to tubulin dimers in PDB-ID: 4DRX and PDB-ID-6GWD.

D) Close-up views of the αβ-tubulin intradimer regions in the interface in TBC-DEG compared to TBC-DEG/TBCC bound states showing changes in residue interaction leading to weakening the αβ-tubulin intradimer in TBC-DEG bound states.

E) Close-up atomic views of the TBC-DEG bound αβ-tubulin intradimer interfaces

F) Close-up atomic views of the TBC-DEG/TBCC bound αβ-tubulin intradimer interface matching views in E.

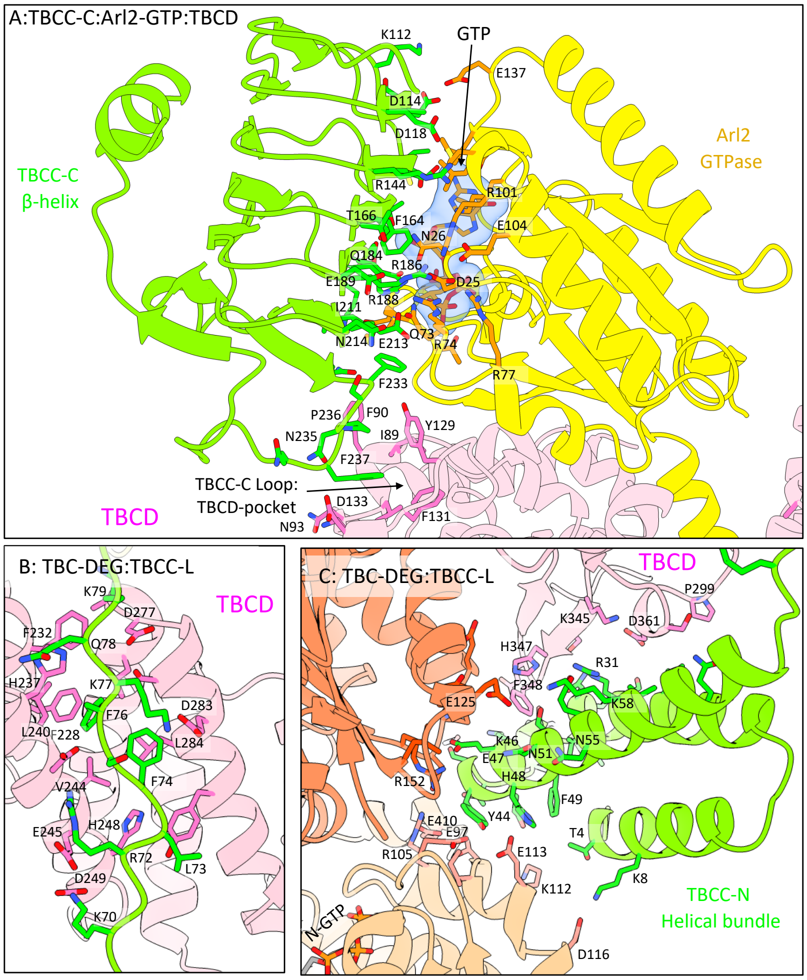

Supplementary Fig 15: Close-up Atomic interfaces of the three TBCC domains with TBC-DEG-αβ-tubulin.

A) An AlphaFold 3 model, as seen in Supplementary Fig 16, based of residue interaction view of the TBCC-C-Arl2 interface. TBCC-C β-helix (light green) and the Arl2 (light orange) are shown, with residues shown in a darker color in stick format with the GTP nucleotide. These interactions match the AlphaFold3 model interfaces as presented in Supplementary Fig 16.

B) Cryo-EM model view of the TBCC-L and TBCD spiral interface is shown with TBCC-L (light green) aromatic Phe and Tyr and positively charged Lys and Arg of TBCC-L (dark green) interacting with hydrophobic Phe, Leu, and acidic Glu Asp residues (dark pink) of TBCD. These interactions match the AlphaFold3 model interfaces (Supplementary Fig 16).

C) Cryo-EM model view of the TBCC-N three-helix bundle (light green) interface with TBCD spiral (light pink) and αβ-tubulin intradimer interface (α-tubulin in light orange and β-tubulin in light red). Interacting Residues are shown in darker colors. The TBCC-N three-helix bundle tip is within 8 Å of the αβ-tubulin N-site GTP. These interactions match the AlphaFold3 model interfaces (Supplementary Fig 16). TBCC-TBCE and Arl2 Sequence conservation, in Supplementary Fig 17-S19, and their plots on the structures, in Supplementary Fig 20, provide additional information.

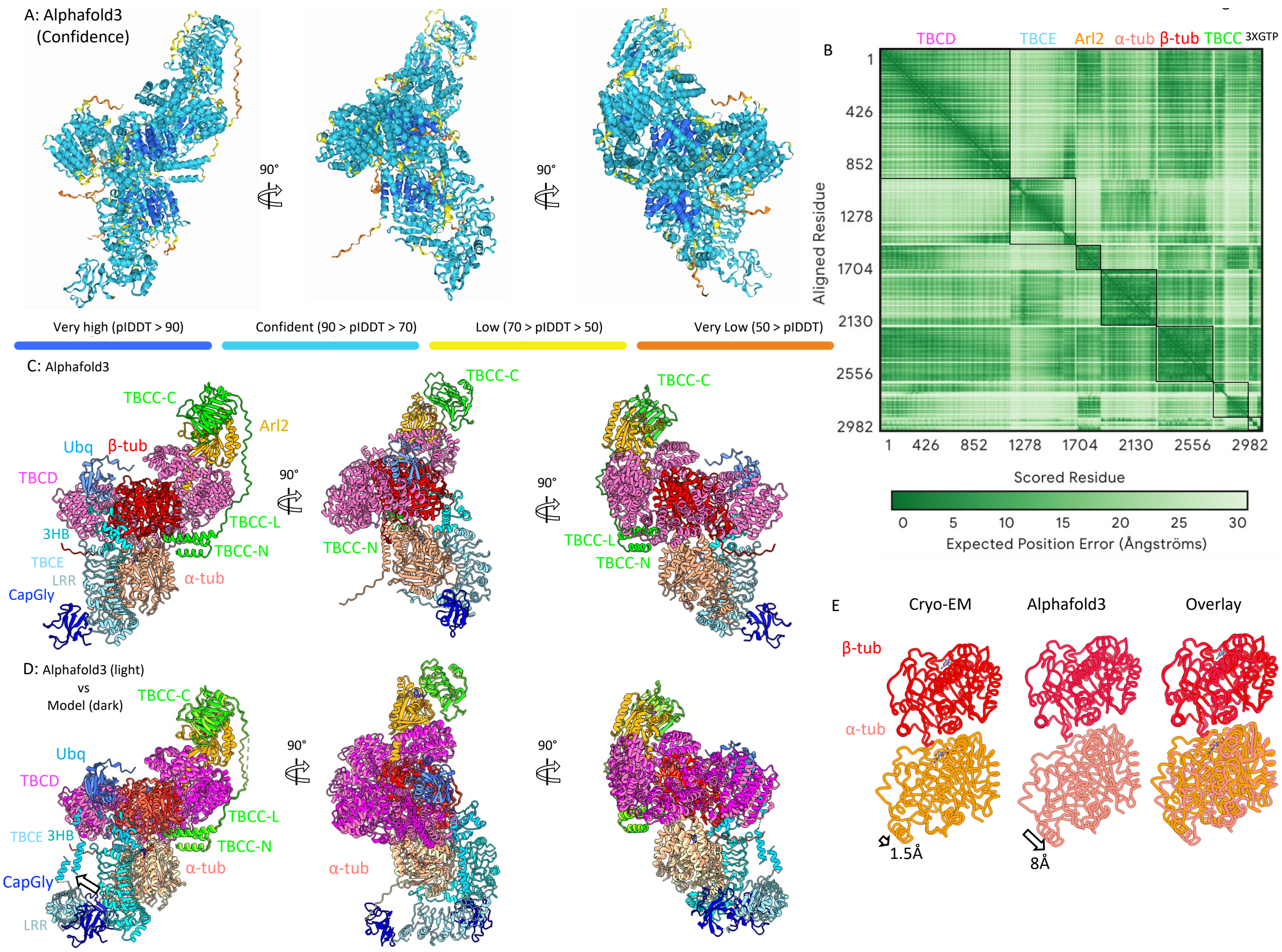

Supplementary Fig 16: AlphaFold3 model for TBC-DEG/TBCC:αβ-tubulin reveals matching TBCC binding interfaces and conformations observed in TBC-DEG:αβ-tubulin cryo-EM model

A) Three rotated views of the TBC-DEG/TBCC-αβ-tubulin AlphaFold3 model in ribbon format colored based on the IDTT confidence scores following the scale in the lower panel

B) Predicted aligned Error (PAE) matrix plot for TBC-DEG/TBCC:αβ-tubulin AlphaFold3 prediction including TBCD, TBCE, Arl2, α- and β-tubulin, TBCC and GTP nucleotides.

C) Three rotated views of the TBC-DEG/TBCC-αβ-tubulin with the subunits and domains colored as shown in Fig. 1.

D) Three rotated views of the superimposed TBC-DEG/TBCC-αβ-tubulin cryo-EM model (dark colors) and AlphaFold3 model (light colors). With regions of difference marked with arrows. Note the complete superimposition of the TBCC-C, TBCC-L and TBCC-N domains (light and dark green), and the match in Arl2 GTPase conformations and differences in the TBCE arm binding to α-tubulin.

E) Comparison of the αβ-tubulin conformations; Cryo-EM model (left), AlphaFold3 (middle) and overlay of both (right).

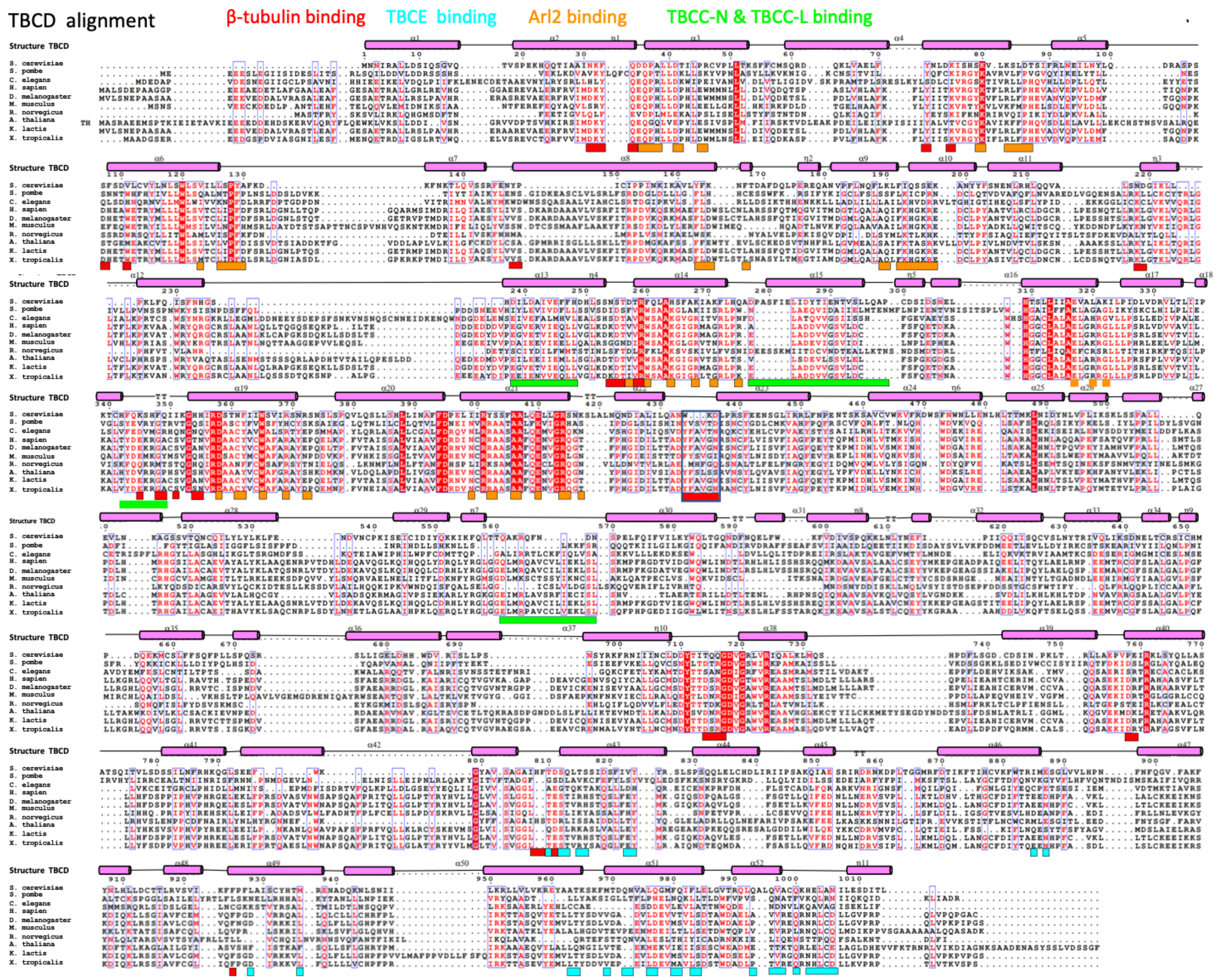

Supplementary Fig 17: **TBCD sequence alignment, secondary structure elements, and subunit interaction sites.** Multiple sequence alignment of TBCD sequences with the secondary structure element boundaries marked on top of the sequences. The interacting residues for each region are marked below with the colored panels of the interacting subunits.

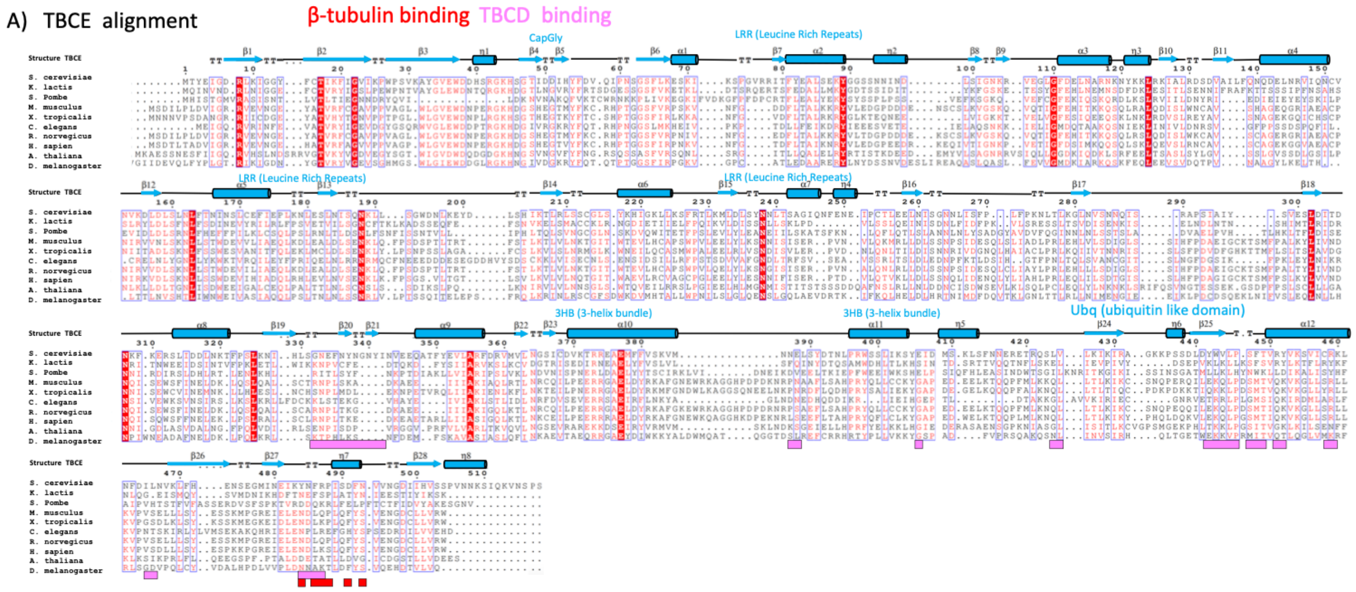

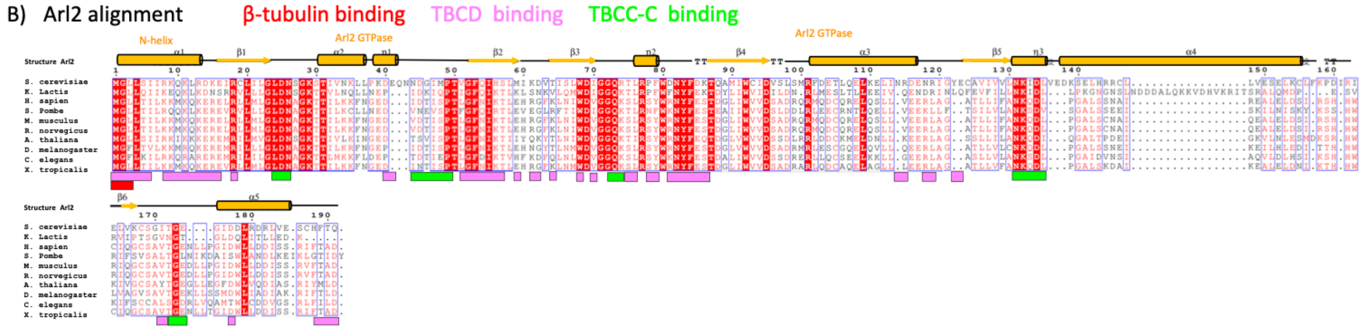

Supplementary Fig 18: **TBCE and Arl2 sequence alignments, secondary structure elements, subunit interaction sites.**

A) Multiple sequence alignment of TBCE orthologs with the secondary structure element boundaries marked on top of the sequences. The interacting residues for each region are marked below with the colored panels of the interacting subunits.

B) Multiple sequence alignment of Arl2 orthologs with the secondary structure element boundaries marked on top of the sequences. The interacting residues for each region are marked below with the colored panels of the interacting subunits.

A: TBCC alignment α-tubulin binding β-tubulin binding TBCD binding Arl2 binding

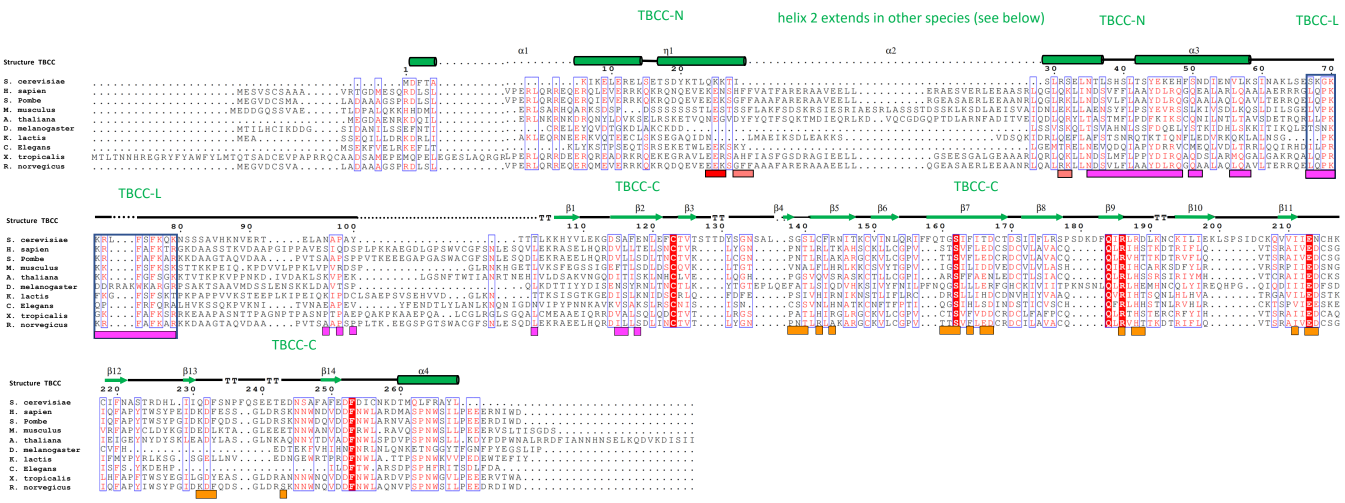

B: Alpha fold 2 models for TBCC orthologs from divergent species demonstrates variation in TBCC-N first helix length

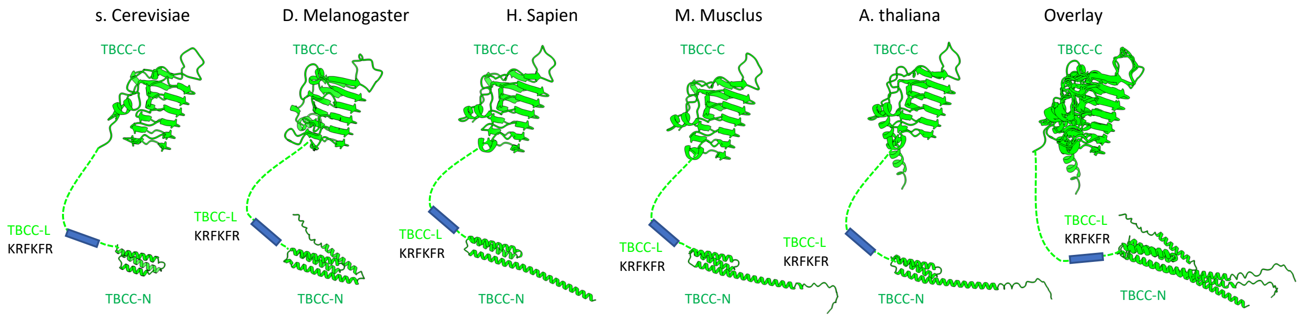

Supplementary Fig 19: **TBCC sequence alignment, structure boundaries, subunit interaction sites, and models for TBCC domains across species showing variations in TBCC-N first helical segment length.**

A) Multiple sequence alignment of TBCC orthologs with the secondary structure element boundaries marked on top of the sequences. The interacting residues for each region are marked below with the colored panels of the interacting subunits.

B) AlphaFold2 prediction models for TBCC orthologs from multiple organisms show the structural conservation in TBCC-N and TBCC-C. TBCC-N helix 1 varies dramatically in length across various species compared to its short length in S. Cerevisiae.

Figure S20

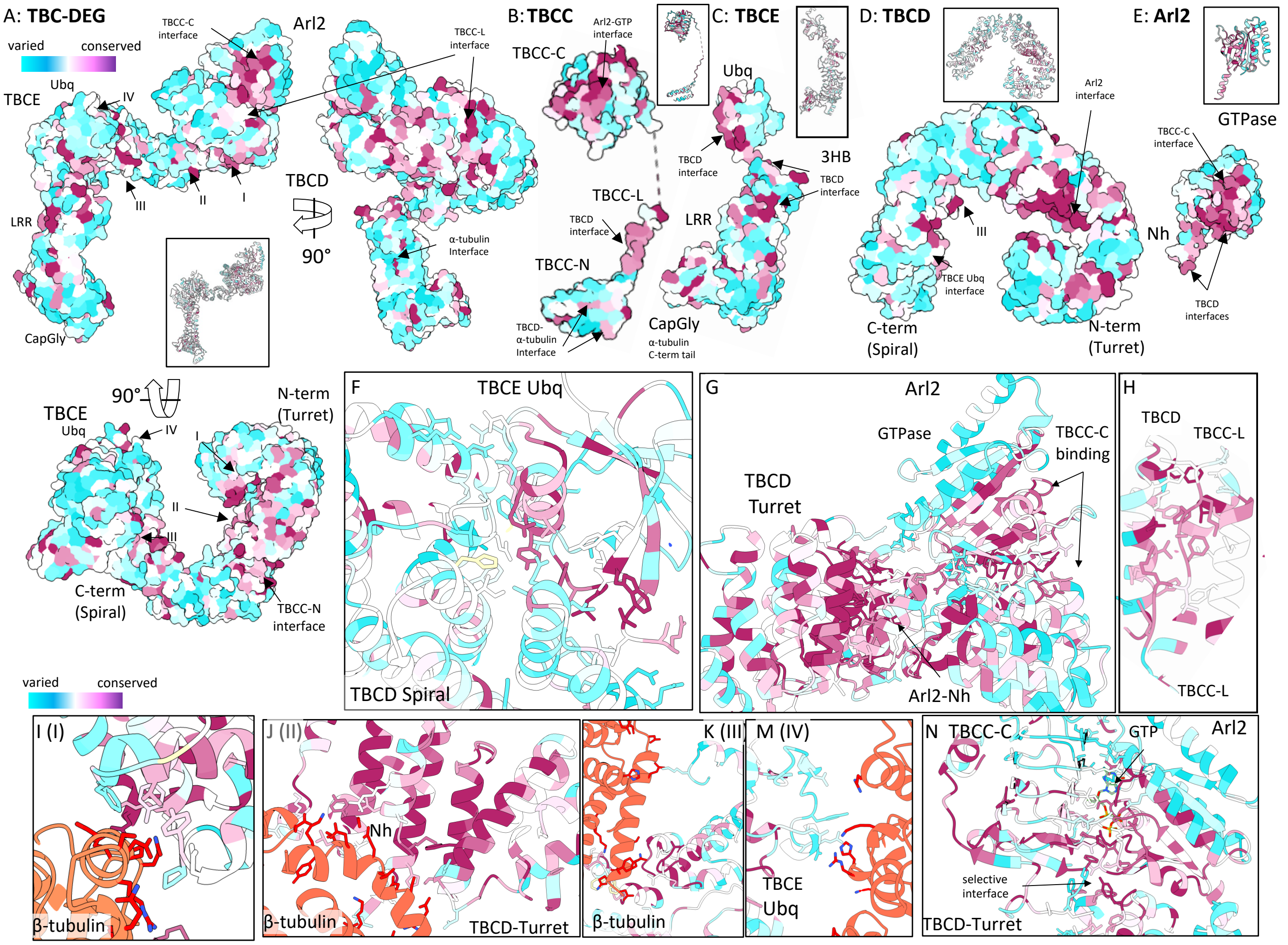

Supplementary Fig 20: **Conserved surfaces of TBC-DEG, TBCD, TBCE, Arl2, and TBCC structures: conservation in interfaces forming the TBC-DEG assembly and interactions with TBCC, and αβ-tubulin subunits**.

A) Three 90°-rotated views of surface representations of the TBC-DEG assembly (αβ-tubulin removed) with the conserved to varied residues colored from purple to blue, based on the scale shown on the top left. The inset shows the structure view in ribbon format. Highlighted regions include: Three TBCD binding interfaces for β-tubulin (I, II, III), TBCE Ubq-β-tubulin interface (IV), TBCC binding interface on the Arl2 GTPase surface, TBCC-L and TBCC-N binding interfaces on TBCD-spiral region

B) A Surface representation of TBCC domains TBCC-N and TBCC-C showing conservation in binding Arl2 interface and TBCC-L and TBCC-N binding interfaces for TBCD spiral domain

C) A Surface representation of TBCE showing surface conservation. The inset shows the structure view in ribbon format. Highlighted are the Ubq domain and 3HB binding interfaces to TBCD.

D) Top view of a surface representation of TBCD showing surface conservation. The inset shows the structure in ribbon format. Highlighted are TBCD surface-conserved interfaces for binding β-tubulin (I, II, III), Arl2, and TBCE.

E) Two 90° views of surface representation of Arl2 showing surface conservation. The inset shows the structure view in ribbon format. Highlighted are the surface-conserved interfaces for TBCD

F) Close-up view of the TBCD Spiral-TBCE Ubq interface with both TBCD and TBCE side chains colored to show conservation, shown in a similar view to Supplementary Fig 5C.

G) Close-up view of the TBCD Turret-Arl2 interface with both TBCD and Arl2 chains colored to show conservation, shown in a similar view to Supplementary Fig 5D.

H) Close up view of the TBCC-L TBCD interface with residues colored for conservation with similar view as shown in Supplementary Fig 15.

I-M) Close-up view of the β-tubulin interfaces with TBCD (I, II, III) and with TBCE-Ubq (IV) interfaces shown in similar views to Supplementary Fig 6D-I, showing the conservation with β-tubulin.

N) Close-up view of the TBCC-C Arl2 GTPase interface with conservation colored for both Arl2 and TBCC subunits.

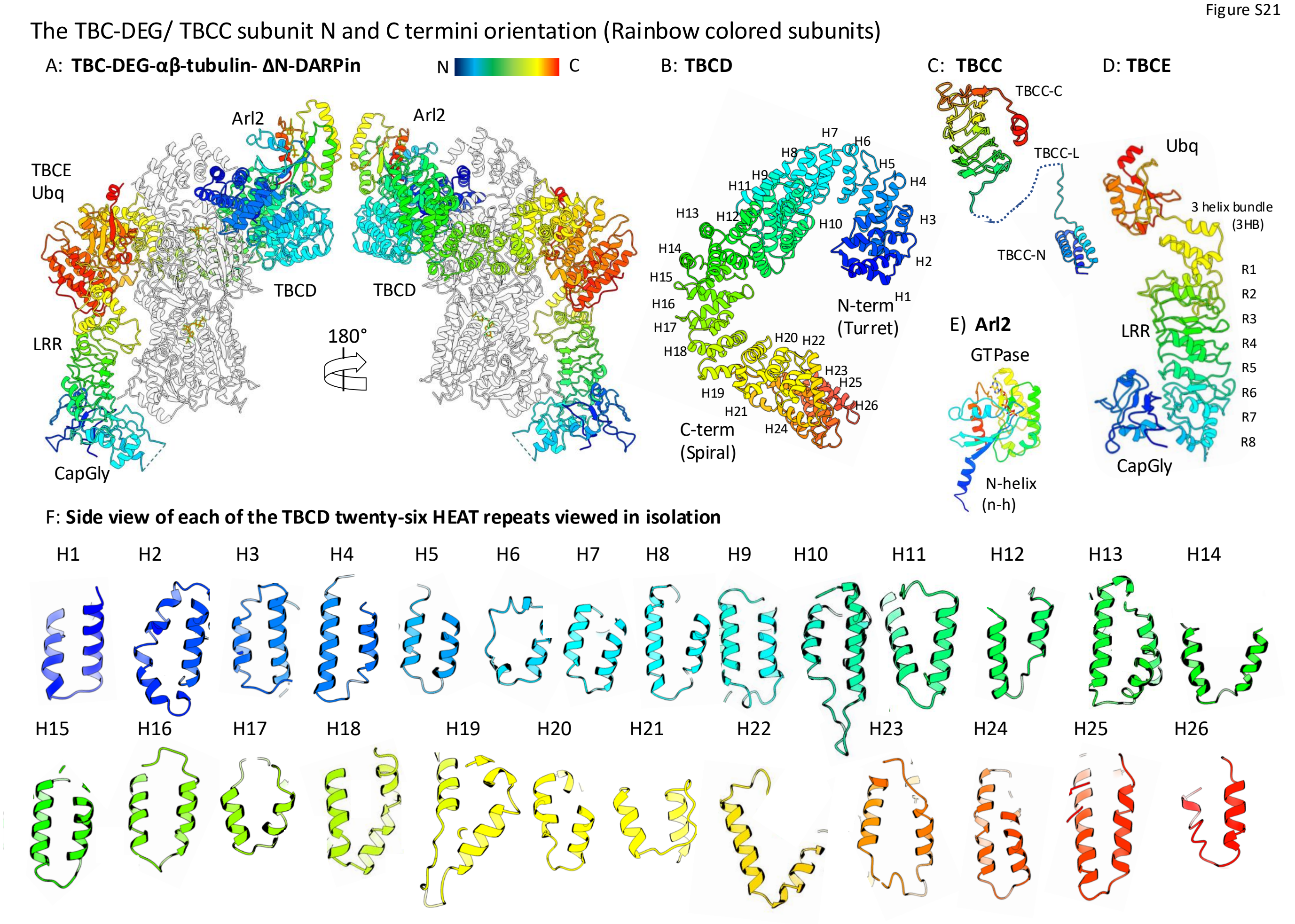

Supplementary Fig 21: Rainbow ribbon representation of TBCD, TBCE, Arl2, and TBCC subunits and their interactions within TBC-DEG and dissociated TBCD HEAT repeats revealing their overall length and organization in TBCD.

A) Two 90°-rotated views of TBC-DEG-αβ-tubulin assembly with αβ-tubulin and ΔN-DARPin are shown in white. TBCD, TBCE, and Arl2 are shown in rainbow-colored ribbon format with a palette described according to the insert.
B) Top view of TBCD in ribbon rainbow color representation with its 26 HEAT repeats marked. Note E shows these HEAT repeat in isolation
C) The side view of TBCC in the rainbow color representation shows its three domains.
D) Side view of TBCE in ribbon rainbow color representation, showing its four domains marked. With LRR repeats marked R1-R8
E) Side view of Arl2 in ribbon rainbow color representation, showing its two domains.
F) Side view of each of the TBCD twenty-six HEAT repeats shown in isolation, side by side, using colors shown in panel B.

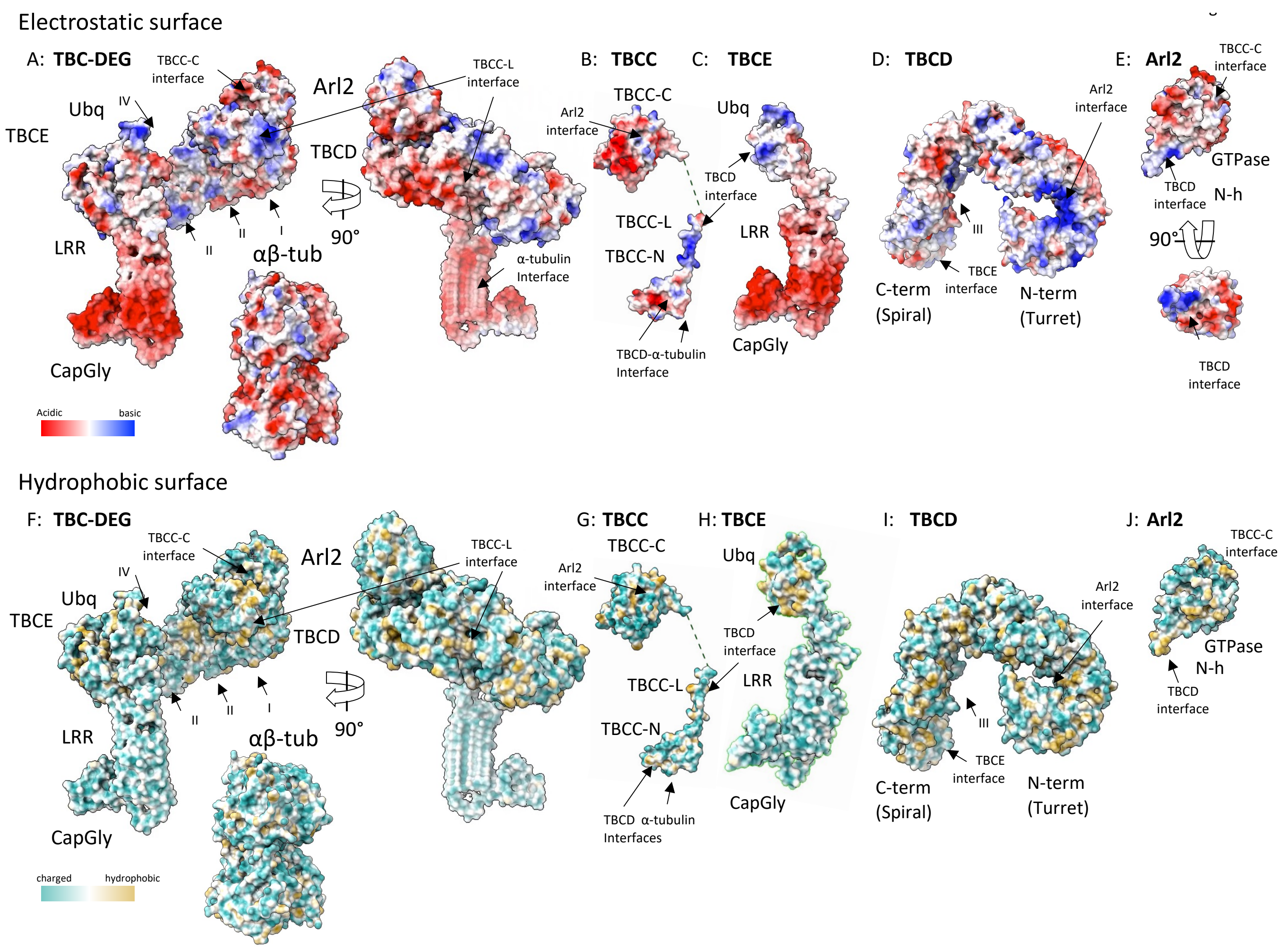

Supplementary Fig 22: **Electrostatic and hydrophobic surface representation of TBC-DEG, TBCD, TBCE, Arl2, and TBCC subunits showing the nature of the charged or hydrophobic nature of these interaction sites.**

A) Two 90°-rotated views of an electrostatic surface representation of the TBC-DEG assembly colored according to the insert shown on the bottom left. The β-tubulin-binding interfaces (I, II, III, IV) and the TBCC-L interface are highlighted. The isolated αβ-tubulin in a similar color representation is also shown in a similar orientation to the left panel.

B) Side-view electrostatic surface representation of TBCC with its three domains shown. The Arl2, TBCD, and α-tubulin interfaces are highlighted.

C) A side-view electrostatic surface representation of TBCE with its four domains is shown. The TBCD, β-tubulin, and α-tubulin interfaces are highlighted.

D) The top-view electrostatic surface representation of TBCD is shown. The β-tubulin (I, II, III), Arl2, and TBCE interfaces are highlighted.

E) Two 90°-rotated electrostatic surface representations of Arl2 with its two domains are shown. The TBCD and TBCC interfaces are highlighted.

F) Two 90°-rotated views of the hydrophobic charged surface representation of the TBC-DEG assembly colored according to the insert shown on the bottom left. The β-tubulin-binding interfaces (I, II, III, IV) and the TBCC-L interface are highlighted. The isolated αβ-tubulin in a similar color representation is also shown in a similar orientation to the left panel.

G) Side-view hydrophobic charged surface representation of TBCC with its three domains shown. The Arl2, TBCD, and α-tubulin interfaces are highlighted.

H) A side-view hydrophobic charged surface representation of TBCE with its four domains is shown. The TBCD, β-tubulin, and α-tubulin interfaces are highlighted.

I) The top-view hydrophobic charged surface representation of TBCD is shown. The β-tubulin (I, II, III), Arl2, and TBCE interfaces are highlighted.

J) Two 90°-rotated hydrophobic charged surface representations of Arl2 with its two domains are shown. The TBCD and TBCC interfaces are highlighted.

Supplementary Reference:

1 Nithianantham, S. *et al.* Tubulin cofactors and Arl2 are cage-like chaperones that regulate the soluble alphabeta-tubulin pool for microtubule dynamics. *Elife* **4** (2015). https://doi.org:10.7554/eLife.08811
