# Supplementary material for: Cryo-EM structures of the tubulin cofactors reveal the molecular basis of alpha/beta-tubulin biogenesis
Source: Nat Commun. 2025 Dec 29;17:1405. doi: 10.1038/s41467-025-68142-0 (PMC12881605; doi:10.1038/s41467-025-68142-0)
Supplement: Supplementary file 2 — Description of Additional Supplementary Files [file 41467_2025_68142_MOESM2_ESM.pdf]

Description of Additional Supplementary Files

Supplementary Movie 1: (Accompanies Fig. 1) TBC-DEG-αβ-tubulin cryo-EM maps, atomic models, and TBCE conformational changes. The Supplementary Movie shows 360° rotation TBC-DEG-αβ-tubulin class 1 cryo-EM map, followed by a map to model view of class 1, TBC-DEG-αβ-tubulin class 2 cryo-EM map, followed by a map to model of class 2, followed by the model transition between state 1 and 2.

Supplementary Movie 2: (Accompanies Fig. 2) Exploring TBC-DEG binding interfaces for αβ-tubulin and interfaces stabilizing TBCD, TBCE, and Arl2 interactions in TBC-DEG. The Supplementary Movie shows a breakout view followed by rotation of TBC-DEG and αβ-tubulin showing the subunit binding footprints colored per subunit.

Supplementary Movie 3: (Accompanies Fig. 3) TBC-DEG/TBCC-αβ-tubulin cryo-EM maps, atomic models, and conformational changes. The movie shows 360° rotation TBC-DEG/TBCC-αβ-tubulin state 1 cryo-EM map, followed by a map to model view of state 1.

Supplementary Movie 4: (Accompanies Fig. 4**)** Exploring TBCC binding interfaces on TBC-DEG-αβ-tubulin. The movie shows a breakout view followed by rotation of TBC-DEG-αβ-tubulin and TBCC showing the subunit binding footprints colored per subunit.
